# Fast segmentation with the NextBrain histological atlas

**DOI:** 10.1162/IMAG.a.1244

**Published:** 2026-05-26

**Authors:** Oula Puonti, Jackson Nolan, Robert Dicamillo, Yael Balbastre, Adria Casamitjana, Matteo Mancini, Eleanor Robinson, Loic Peter, Roberto Annunziata, Juri Althonayan, Shauna Crampsie, Emily Blackburn, Benjamin Billot, Alessia Atzeni, Peter Schmidt, James Hughes, Jean C. Augustinack, Brian L. Edlow, Lilla Zöllei, David L. Thomas, Dorit Kliemann, Martina Bocchetta, Catherine Strand, Janice L. Holton, Zane Jaunmuktane, Juan Eugenio Iglesias

**Affiliations:** Danish Research Centre for Magnetic Resonance, Centre for Functional and Diagnostic Imaging and Research, Copenhagen University Hospital – Amager and Hvidovre, Copenhagen, Denmark; Athinoula A. Martinos Center for Biomedical Imaging, Massachusetts General Hospital and Harvard Medical School, Boston, MA, United States; Department of Medical Physics and Biomedical Engineering, University College London, London, United Kingdom; Department of Experimental Psychology, University College London, London, United Kingdom; Research Institute of Computer Vision and Robotics, University of Girona, Girona, Spain; Enrico Fermi Research Center, Rome, Italy; Cardiff University Brain Research Imaging Centre, Cardiff University, Cardiff, United Kingdom; Computer Science and Artificial Intelligence Laboratory, Massachusetts Institute of Technology, Cambridge, MA, United States; Advanced Research Computing Centre, University College London, London, United Kingdom; Center for Neurotechnology and Neurorecovery, Department of Neurology, Massachusetts General Hospital and Harvard Medical School, Boston, MA, United States; Dementia Research Centre, Department of Neurodegenerative Disease, UCL Queen Square Institute of Neurology, University College London, London, United Kingdom; Neuroradiological Academic Unit, Department of Translational Neuroscience and Stroke, UCL Queen Square Institute of Neurology, University College London, London, United Kingdom; Department of Psychological and Brain Sciences, University of Iowa, Iowa City, IA, United States; Centre for Cognitive and Clinical Neuroscience, Division of Psychology, Department of Life Sciences, College of Health, Medicine and Life Sciences, Brunel University of London, London, United Kingdom; Queen Square Brain Bank for Neurological Disorders, Department of Clinical and Movement Neurosciences, UCL Queen Square Institute of Neurology, University College London, London, United Kingdom

**Keywords:** brain MRI segmentation, histological atlas, domain-agnostic neuroimaging

## Abstract

Structural brain analysis at the subregion level offers critical insights into healthy aging and neurodegenerative diseases. The NextBrain histological atlas was recently introduced to support such fine-grained investigations, but its existing Bayesian segmentation framework remains computationally prohibitive, particularly for large-scale studies. We present a new, open-source tool that dramatically accelerates segmentation using a hybrid approach combining: machine learning, contrast-adaptive segmentation; target-specific image synthesis; and fast diffeomorphic registration (all three with GPU support). Our method enables highly granular segmentation of brain MRI scans of any resolution and contrast (*in vivo or ex vivo*) at a fraction of the computational cost of the original method (<5 minutes on a GPU). We validate our tool on four different modalities (*in vivo* MRI, *ex vivo* MRI, HiP-CT, and photography) across a total of approximately 4,000 brain scans. Our results demonstrate that the accelerated approach achieves comparable accuracy to the original method in terms of Dice scores, while reducing runtime by over an order of magnitude. This work enables high-resolution anatomical analysis at unprecedented scale and flexibility, providing a practical solution for large neuroimaging studies. Our tool is publicly available in FreeSurfer (https://surfer.nmr.mgh.harvard.edu/fswiki/HistoAtlasSegmentation).

## 1 Introduction

Image segmentation is a fundamental component of human neuroimaging. Automatic delineation of anatomical structures on MRI scans is critical for quantifying volumes of regions of interest (ROIs), assessing their morphometry, and supporting downstream analyses such as functional localization or connectivity modeling. Structural segmentation facilitates the study of brain development (Giedd et al., 1999; Gogtay et al., 2004), aging (Bethlehem et al., 2022), and various neurological and psychiatric disorders (Jack Jr et al., 1999), and is routinely used to derive imaging biomarkers for conditions such as Alzheimer’s disease, schizophrenia, and multiple sclerosis (Cuingnet et al., 2011; Dickerson et al., 2011; Frisoni et al., 2010).

As the scale and diversity of neuroimaging datasets increase, robust and generalizable segmentation tools are needed to handle variation in acquisition protocols, scanner vendors, and patient populations. This is particularly important when sharing open-source tools (as opposed to developing in-house solutions), since few assumptions can be made on the MRI contrast properties of the input scans. For this reason, Bayesian segmentation methods based on generative models (Ashburner & Friston, 2005; Pohl et al., 2006; Puonti et al., 2016; Van Leemput et al., 2002; Wells et al., 1996; Zhang et al., 2002) have long been central to widespread neuroimaging software pipelines such as SPM (Friston et al., 1994), FreeSurfer (Fischl, 2012), FSL (Smith et al., 2004), or AFNI (Cox, 1996).

In terms of generalizability, a particularly interesting subclass of Bayesian segmentation methods uses unsupervised likelihood models, which makes it adaptive to MRI contrast. Best represented by Unified Segmentation (Ashburner & Friston, 2005) (distributed with SPM), these methods combine supervised anatomical priors (probabilistic atlases of anatomy) with unsupervised likelihoods that typically comprise a Gaussian mixture model (GMM) and a bias field model. Crucially, the parameters of the GMM and the bias field model are estimated directly from the input scan, which can thus have been acquired with any pulse sequence. Furthermore, because the models of anatomy and image formation are decoupled, high-resolution *ex vivo* data (e.g., histology, *ex vivo* MRI) can be leveraged to construct detailed atlases, which can subsequently be applied to the segmentation of markedly different, lower-quality *in vivo* images. We have successfully used *ex vivo* MRI and histology to build probabilistic atlases of the hippocampus (Iglesias et al., 2015), amygdala (Iglesias et al., 2017), and thalamus (Iglesias et al., 2018) at the subregion level, which can be flexibly applied to *in vivo* segmentation.

Our group has recently extended *ex vivo* atlasing from a couple of brain structures to the whole human brain using histology (Casamitjana et al., 2025). Our new atlas (“NextBrain”) comprises 333 ROIs per hemisphere, defined at 200 µm resolution. NextBrain includes a Bayesian segmentation framework capable of transferring this anatomical detail to *in vivo* MRI images. However, the original implementation is computationally intensive: segmenting a single scan at 300 µm resolution can take *days* on a standard CPU-based workstation. While GPU acceleration can reduce run time, it requires access to high-end hardware that is not available to many users, limiting the practicality of the tool for large-scale studies or clinical applications.

One potential solution to this problem would naturally be deep learning, which provides fast inference times and impressive results in a variety of medical image segmentation tasks (Isensee et al., 2021; Ronneberger et al., 2015), including neuroimaging (Henschel et al., 2020; Pereira et al., 2016; Salehi et al., 2017). Compared with Bayesian segmentation, these approaches suffer from “domain shift”, that is, they often struggle to generalize to unseen domains without careful finetuning or domain adaptation (Guan & Liu, 2021; Wang & Deng, 2018). There have been attempts to address this domain shift problem; one representative example for brain MRI segmentation is SynthSeg (Billot et al., 2023), which relies on domain randomization. Its design is directly inspired by Bayesian segmentation, and it relies on generating synthetic images with almost the same generative model. Importantly, these images are synthesized with random model parameters to simulate broad variations in image appearance, which leads to high robustness at test time. Despite these advances, current deep learning approaches remain limited in transferring high-resolution information from *ex vivo* domains to *in vivo* data, particularly when only sparse ground-truth annotations are available across a large number of ROIs—as in NextBrain.

In this work, we present a fast and robust reimplementation of Bayesian segmentation that enables segmentation with NextBrain in practical times without specialized hardware. Our method leverages carefully chosen approximations, recent advances in deep learning for initialization, contrast-adaptive synthetic image generation, and fast diffeomorphic registration to deliver highly detailed segmentations of *in vivo* and *ex vivo* brain MR scans with minimal computational demands. Crucially, the pipeline runs without any manual parameter tuning and achieves performance comparable to the original implementation, while reducing run time by more than an order of magnitude. This contribution makes detailed brain parcellation accessible to a wider community and enables high-resolution neuroanatomical analysis at scale.

## 2 Methods

### 2.1 Bayesian segmentation and NextBrain

#### 2.1.1 Preliminaries

Bayesian segmentation is typically formulated as an inference problem within a generative model of the data:

$$\hat{L}=\underset{L}{\text{arg max}}p(L|I)=\underset{L}{\text{arg max}}p(I|L)p\left(L\right),$$

where L denotes the label assignment (i.e., the segmentation), and I represents the observed image. In general, both the likelihood p(I|L) and the prior p(L) are governed by parameter sets, denoted $\theta_{I}$ and $\theta_{L}$, respectively. Exact posterior inference requires marginalization over these parameters, which is intractable in practice. A common approximation is to compute point estimates of the parameters by maximizing their posterior distribution:

$$\begin{array}{c} \left\{{\hat{\theta }}_{I},{\hat{\theta }}_{L}\right\}=\underset{\theta_{I},\theta_{L}}{\text{arg max}}p(\theta_{I},\theta_{L}|I)\\ =\underset{\theta_{I},\theta_{L}}{\text{arg max}}p\left(\theta_{I}\right)p\left(\theta_{L}\right)\underset{L}{\sum }\;p(I|L,\theta_{I})p(L|\theta_{L}), \end{array}$$ (1)

where we have made the standard assumption that the priors over the two parameter sets are independent, that is, $p\left(\theta_{I},\theta_{L}\right)=p\left(\theta_{I}\right)p\left(\theta_{L}\right)$. Given the estimated parameters, the final segmentation is computed by maximizing the posterior over labelings:

$$\hat{L}=\underset{L}{\text{arg max}}p(L|I,{\hat{\theta }}_{I},{\hat{\theta }}_{L})=\underset{L}{\text{arg max}}p(I|L,{\hat{\theta }}_{I})p(L|{\hat{\theta }}_{L}).$$ (2)

#### 2.1.2 Model instantiation in NextBrain

NextBrain (Casamitjana et al., 2025) uses a generative model similar in structure to Unified Segmentation (Ashburner & Friston, 2005) and SAMSEG (Puonti et al., 2016), which we briefly summarize here. The prior is defined by a probabilistic atlas A, which specifies conditionally independent categorical distributions over K possible labels at each spatial location. This atlas is deformed using a nonlinear transformation ϕ, parameterized by $\theta_{L}$. The deformation follows a probabilistic model encoded by a prior distribution $p\left(\theta_{L}\right)$, which penalizes excessive geometric distortion via a membrane energy term. Specifically, the probability distribution over the labeling L is defined as:

$$p(L|\theta_{L})=\prod_{j=1}^{J}p(L_{j}|A\left(\phi \left(x_{j},y_{j},z_{j};\theta_{L}\right)\right),$$ (3)

where j indexes the J voxels in the image, and $\left(x_{j},y_{j},z_{j}\right)$ represents the spatial coordinates of voxel j. The prior over the deformation parameters $\theta_{L}$ is given by:

$$p\left(\theta_{L}\right)\propto \text{exp}\left[-\lambda E_{\text{mem}}\left(\phi \left(\theta_{L}\right)\right)\right],$$ (4)

where λ is a regularization constant, and $E_{\text{mem}}$ denotes the membrane energy, defined as:

$$\begin{array}{l} E_{\text{mem}}\left(\theta_{L}\right)\\ \;=\sum_{j=1}^{J}\sum_{i=\left(x,y,z\right)}{\left[{\left(\frac{\partial \phi_{i}}{\partial x}\right)}^{2}+{\left(\frac{\partial \phi_{i}}{\partial y}\right)}^{2}+{\left(\frac{\partial \phi_{i}}{\partial z}\right)}^{2}\right]}_{\left(x=x_{j},y=y_{j},z=z_{j}\right)} \end{array}$$

Given a labeling L, the likelihood models the image intensities using a GMM, assuming conditional independence across voxels and incorporating a smoothly varying multiplicative bias field. To simplify the model, the image intensities I are log-transformed, so that the bias becomes additive and the need to rescale probability densities due to multiplicative bias is eliminated. The likelihood is given by:

$$p(I|L,\theta_{I})=\prod_{j=1}^{J}g\left(I_{j}-\sum_{b=1}^{B}c_{b}\Psi_{b}\left(x_{j},y_{j},z_{j}\right);\mu_{L_{j}},\sigma_{L_{j}}\right),$$ (5)

where g(⋅;μ,σ) denotes the probability density function of a Gaussian distribution with mean μ and standard deviation σ, and ${\left\{c_{b}\right\}}_{b=1}^{B}$ are the coefficients of the bias field basis functions ${\left\{\Psi_{b}\right\}}_{b=1}^{B}$. The full set of likelihood parameters is given by $\theta_{I}=\left\{\mu_{1},\ldots ,\mu_{K},\sigma_{1},\ldots ,\sigma_{K},c_{1},\ldots ,c_{B}\right\}$, which includes class-specific means and variances, along with the bias field coefficients. A non-informative (uniform) prior is assumed over $\theta_{I}$, such that no specific MRI contrast or bias field is favored: $p\left(\theta_{I}\right)\propto 1$ .

#### 2.1.3 Segmentation as Bayesian inference

Substituting the prior and likelihood models (Eqs. (3), (4), and (5)) into Eq. (1), taking logarithm, and disregarding constant terms that do not affect the optimization, the following objective function is obtained:

$$\begin{array}{lll} ℒ\left(\theta_{I},\theta_{L}\right)=\sum_{j=1}^{J}\;\text{log}[\overset{K}{\underset{k=1}{\sum \;}}p\left(k\text{|}A\left(\phi \left(x_{j},y_{j},z_{j};\theta_{L}\right)\right)g\left(I_{j}-\sum_{b=1}^{B}\;c_{b}\Psi_{b}\left(x_{j},y_{j},z_{j}\right);\mu_{k},\sigma_{k}\right)\right] & & \\ \;\;\;\;\;\;-\lambda E_{\text{mem}}\left(\phi \left(x,y,z;\theta_{L}\right)\right). & & \end{array}$$ (6)

**Fig. 1.**
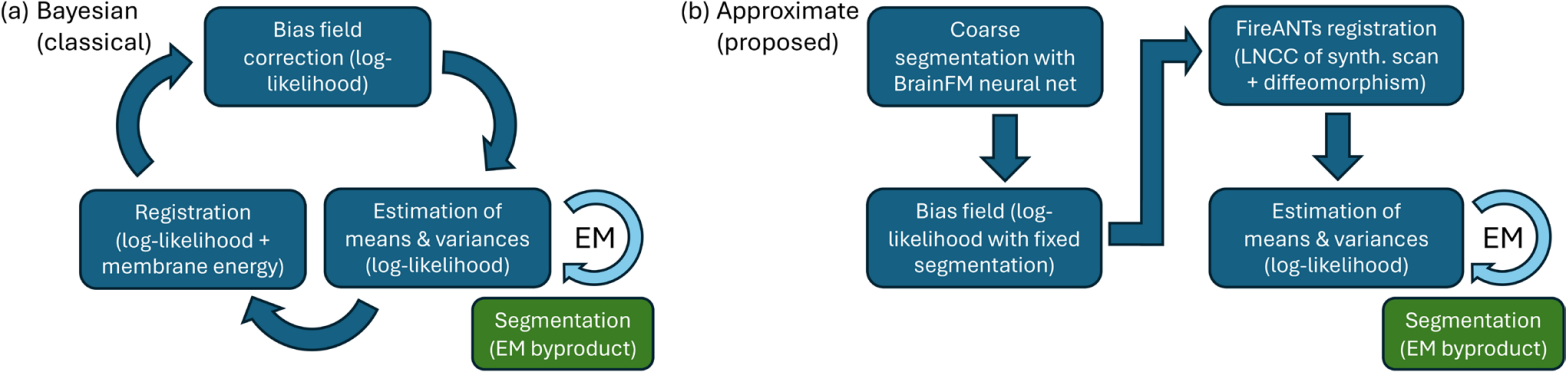
(a) Traditional Bayesian segmentation iteratively updates bias field parameters, Gaussian parameters, and registrations parameters. Furthermore, updating the Bayesian parameters has a nested loop of updates with the Expectation Maximization (EM) algorithm; segmentation is obtained as a by-product of EM. (b) Our proposed algorith is approximate but much faster, as it visits each set of parameters only once.

This objective function is maximized using a coordinate ascent scheme, in which the parameters $\theta_{L}$ and $\theta_{I}$ are alternately optimized (Fig. 1a). The prior parameters $\theta_{L}$ (representing the atlas deformation) are updated using standard numerical optimization methods (Nocedal & Wright, 2006). The likelihood parameters $\theta_{I}$ are optimized using the generalized Expectation Maximization (GEM) algorithm (Dempster et al., 1977). GEM alternates between constructing a lower bound on the objective ℒ via Jensen’s inequality, and maximizing this bound with respect to $\theta_{I}$. This iterative process guarantees an increasing sequence of values for the original objective ℒ.

Maximizing the lower bound involves iteratively updating the means, variances, and bias field parameters. All of these can be updated in closed form given the others. After convergence of this coordinate ascent procedure, the estimated parameters$,{\hat{\theta }}_{L}$ and ${\hat{\theta }}_{I}$ are used to compute the final segmentation, as described in Eq. (2). In practice, explicit computation of this equation is unnecessary, since voxel-wise posterior probabilities over labels (also known as “responsibilities”) are already computed as part of the GEM algorithm. These responsibilities are used in the E-step to construct the lower bound. For further details on this framework, the reader is referred to Ashburner and Friston (2005) or Puonti et al. (2016).

#### 2.1.4 Computational bottlenecks

The original inference algorithm in NextBrain is computationally intensive and often impractical for routine use. Its complexity arises from a deeply nested optimization structure at three levels: *(i)* alternating between deforming the atlas and updating intensity model parameters; *(ii)* the latter uses a GEM algorithm that iterates between E and M steps; and *(iii)* the M-step itself involves iterative updates of class means, variances, and bias field coefficients, each depending on the others.

NextBrain’s nested optimization structure results in long run times, especially when combined with its large number of anatomical labels and high spatial resolution. NextBrain includes 333 ROIs per hemisphere, which are coarsely grouped during inference but ultimately expanded to the full label set when computing the final segmentation. The atlas is defined at 0.2 mm isotropic resolution, that is, 125 times more voxels than a standard 1 mm MRI. Even at the default working resolution of 0.3 mm, voxels are 37 times smaller. Although resolution could be further reduced to alleviate computational requirements, this would compromise the benefits of a high-resolution histological atlas.

The combination of model complexity, label granularity, and high spatial resolution results in substantial computational and memory demands. Segmenting a single case at 0.3 mm resolution takes over 48 hours on a CPU and still requires about 60 minutes on a GPU with at least 24 GB of memory. *Ex vivo* segmentation at 0.2–0.3 mm resolution is often infeasible with the current implementation. This large computational footprint limits the practicality of NextBrain as a segmentation tool. In the next section, we present a reimplementation that preserves the advantages of using our high-resolution atlas while dramatically improving run time and hardware efficiency through targeted approximations and algorithmic redesign.

### 2.2 Approximate inference for fast segmentation

#### 2.2.1 Overview

Here, we present a fast segmentation method for NextBrain (Fig. 1b) that efficiently approximates the inference problem defined in Eq. (1). Unlike traditional approaches (1a), our algorithm visits each set of model parameters only once (1b), using GPU-enabled machine learning and a custom GPU-accelerated diffeomorphic registration algorithm. Importantly, the method preserves robustness to variations in MRI modality and contrast by maintaining contrast-adaptive modeling of image intensities. Although the registration involves several hyperparameters, our publicly available implementation uses a fixed set of defaults (employed in all experiments) that do not require manual tuning.

#### 2.2.2 Preprocessing

The input scan is first segmented using the segmentation module of our foundation model “BrainFM” (Liu et al., 2025). This method closely follows SynthSeg (Billot et al., 2023), and produces coarse segmentations at 1 mm resolution that are highly robust to variations in contrast, resolution, and imaging artifacts (including strong bias fields). Compared to the original SynthSeg model, BrainFM adds several labels, including five contralateral ROIs in the limbic system (Greve et al., 2021) and 12 extracerebral structures (see Liu et al., 2025 for details).

These coarse segmentations are used to exclude non-brain tissue, including cerebrospinal fluid (CSF), which NextBrain models with the same class as the background. The segmentation is also used to divide the brain into hemispheres. Non-hemispheric ROIs such as the brainstem and white matter lesions are also split by thresholding the left-right component of a deformation field mapping the scan to a symmetric MNI atlas; this deformation is computed within BrainFM using the Registration-by-Regression (RbR) algorithm (Gopinath et al., 2024; Hu et al., 2025). In addition to preprocessing, the ROI segmentations also play a key role when estimating model parameters in later stages of the pipeline, as described below.

#### 2.2.3 Grouping ROIs

In practice, Bayesian segmentation algorithms group ROIs into a smaller number of tissue classes during inference, clustering regions expected to share similar intensity profiles (e.g., gray matter structures such as the cerebral cortex, hippocampus, and amygdala). This strategy improves robustness, especially for small regions that may otherwise yield noisy parameter estimates. For instance, since the third ventricle is a thin layer of cerebrospinal fluid, its intensity distribution can be more reliably estimated when grouped with the much larger lateral ventricles. Grouping also benefits bias field estimation by signaling that some ROIs should have similar intensity profiles, despite potentially being distant from each other (e.g., left and right putamen); this information greatly helps disambiguate intensity variations due to bias field and anatomy.

By default, our software groups the NextBrain ROIs into 16 tissue classes: cerebral white matter, cerebral gray matter, cerebellar white matter, cerebellar cortex, caudate, putamen, pallidum, lateral thalamus, medial thalamus, red nucleus, compact brainstem white matter, diffuse brainstem white matter, hypothalamus, mammillary bodies, dentate nucleus of the cerebellum, and hippocampal white matter. This default grouping is based on our experience with MRI, Bayesian segmentation, and the NextBrain atlas. Nevertheless, users can specify their own grouping, for example, adding a class to model a tissue type that is visible in a certain imaging modality, or collapsing classes in order to enhance robustness in modalities with limited contrast. Practical details are provided in Section 4 below.

#### 2.2.4 Optimizing the bias field parameters $\left\{{\text{c}}_{\text{b}}\right\}$

We model the bias field with a 3D discrete cosine transform (DCT) basis of order up to 6, resulting in 343 basis functions. To estimate the bias field parameters $\left\{c_{b}\right\}$, we first extract the whole brain using the available segmentation and group the BrainFM labels into eight tissue classes: cerebral gray matter, cerebellar gray matter, cerebral white matter, cerebellar white matter, brainstem, cerebrospinal fluid (CSF), thalamus, and pallidum. A soft segmentation is then generated by blurring the one-hot encoding of this 8-class map with a Gaussian kernel (σ=0.5mm ). This soft segmentation is used to perform a *single M-step* of the GEM algorithm, with responsibilities fixed to the soft assignments. Due to the high reliability of the SynthSeg-based segmentation, the large number of voxels available, and the relatively low number of parameters, reclassifying voxels in an EM loop offers negligible benefit. While the M-step entails a nested optimization that alternates between estimating tissue class statistics (means and variances) and updating the bias field coefficients, convergence is rapid.

#### 2.2.5 Optimizing the prior parameters (atlas registration) $\theta_{\text{L}}$

In Bayesian segmentation, updating the atlas deformation corresponds to optimizing the objective in Eq. (6) with respect to the deformation parameters $\theta_{L}$, while holding the intensity model parameters $\theta_{I}$ fixed. This step amounts to a deformable image registration problem, where the data term is given by the log-likelihood of the image under the atlas and the current estimate of $\theta_{I}$. However, this registration becomes computationally expensive in the classical coordinate ascent scheme, as every $\theta_{I}$ update effectively changes the objective function with respect to $\theta_{L}$, making the optimization slow.

**Fig. 2.**
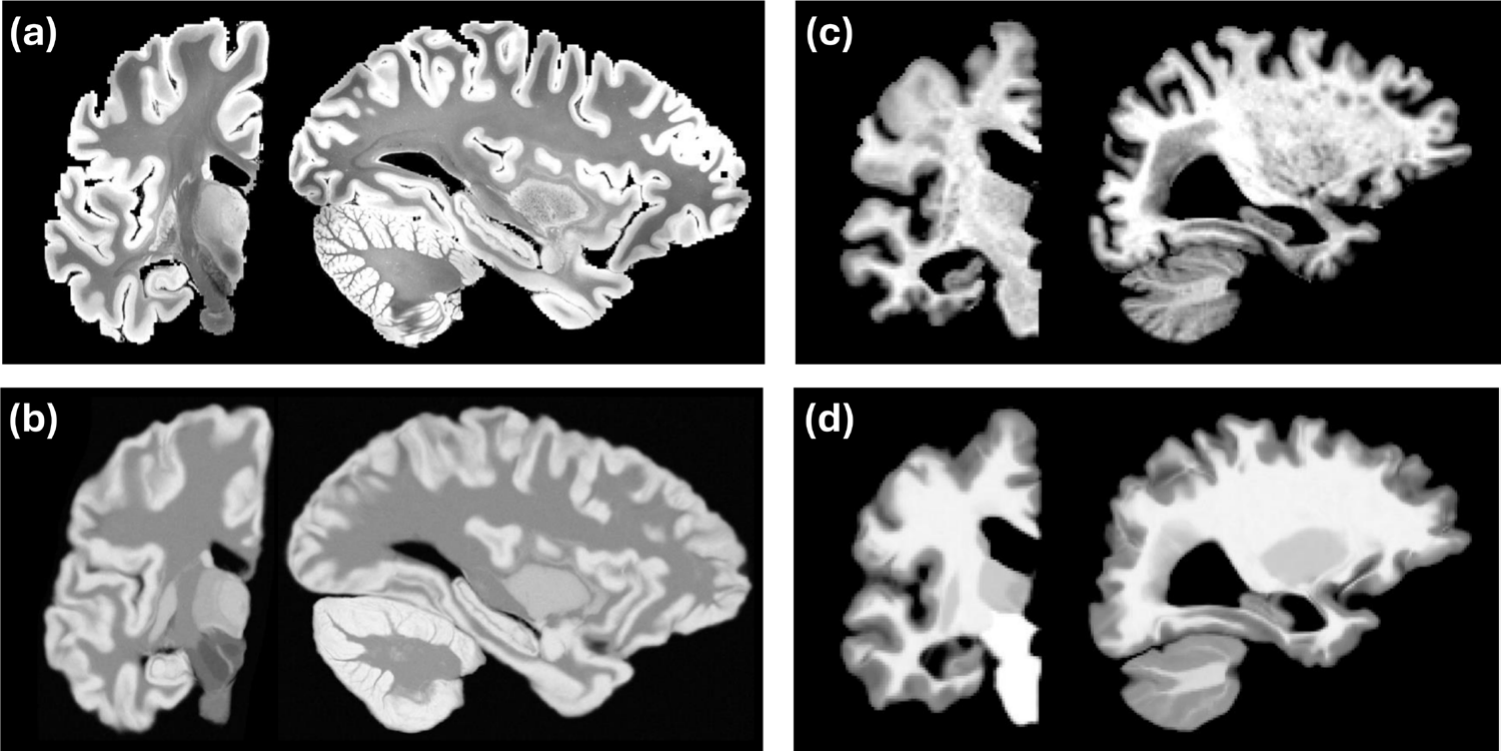
(a) Coronal and sagittal slices of an *ex vivo* scan at 0.2 mm resolution (Edlow et al., 2019). (b) Matching synthetic anatomical volume after registration with FireANTs and composite similarity metric. (c) Slices of a 1 mm isotropic, skull-stripped, T1-weighted scan from the MIRIAD dataset (Malone et al., 2013). (d) Registered synthetic anatomical volume. Note the differences in contrast and resolutions of the synthetic anatomical volumes (c, d), which attempt to match the scans they seek to segment.

To avoid this costly alternation, we leverage the available BrainFM segmentation to bypass the need to update $\theta_{I}$ iteratively. Despite its lower resolution compared to the NextBrain atlas, the BrainFM segmentation is sufficiently accurate to estimate class-wise means and variances from the bias field corrected image using robust statistics: the median intensity of each label is used for the mean, and 1.4826 times the median absolute deviation is used for the standard deviation. We use these parameters to synthesize a Gaussian image (henceforth “synthetic anatomical volume”) from the ROI-grouped probabilistic atlas, which is then resampled to match the resolution of the input scan (Fig. 2) using the model in our previous publication (Billot et al., 2020). The mean intensities for these groups are derived from the BrainFM segmentation using heuristic rules the exploit label correspondences. The default rules (which match the 16-class grouping) are listed in the Supplementary Material, but the user can also specify their own set, for example, to exploit prior knowledge on the intensities or to support a non-default ROI grouping strategy (details in Section 4). This procedure ensures that the synthetic anatomical volume exhibits contrast and resolution resembling those of the target scan, thereby greatly facilitating registration in the subsequent steps described below.

We perform registration between the synthetic anatomical volume and the input image one hemisphere at a time, as NextBrain is a single-hemisphere model. Registration is performed using FireANTs (Jena et al., 2024), which we run in greedy, non-symmetric mode (i.e., with the loss computed in the fixed image grid). The similarity metric comprises two terms: *(i)* local normalized cross-correlation (LNCC, Avants et al., 2008), computed with a kernel radius of 3 voxels; and *(ii)* the mean Dice score between the BrainFM segmentation and the warped atlas labels, where the atlas ROIs are grouped to match the BrainFM protocol. We note that this grouping scheme is nonadjustable and completely independent of the one used to model intensities, since it relies on semantic correspondance of labels. We further note that the intensity term (LNCC), which captures semi-local correlations, renders the intensity-based term robust to residual bias fields and to imperfections in the synthetic anatomical volume model. This robustness explains why the heuristics described above are sufficient in practice to achieve accurate registration across a wide range of resolutions and imaging modalities, including non-MRI modalities. Additional robustness is, of course, provided by the segmentation term. The optimization is carried out using the Adam optimizer (Kingma & Ba, 2014), and proceeds in a multiscale fashion, from coarse to fine resolution levels.

While FireANTs does not explicitly include a regularization term in the objective function, smoothness is implicitly enforced through the multiscale optimization scheme and the use of diffeomorphic transformations parameterized in a smooth velocity field space. This setup discourages implausible deformations by naturally limiting high-frequency updates, ensuring anatomically consistent mappings without the need for hand-tuned regularization weights. We used Gaussian kernels with σ=0.25 and σ=1.0 to smooth the field and its gradient, respectively. These values are fairly liberal and enable the atlas to cope with very strong deformations, as shown in the Results section; the user can modify them if they want, for example, to use more conservative values to analyze a young healthy population (see Section 4). We also note that the classical Bayesian method in Casamitjana et al. (2025) used an explicit regularizer based on the membrane energy (Eq. (4)) of a field parameterized by B-Splines, thus leading to a different distribution of (also smooth) deformation fields; an example is shown in Figure S1 in the Supplementary Material. Examples of brain scans of different contrast and resolution and their corresponding registered synthetic anatomical volumes are shown in Figure 2.

The registration parameters can be adjusted by the user to finetune the registration for specific datasets, but the default parameters work well across resolution and contrast, as shown in Section 3 below. These were chosen as follows. The multi-resolution pyramid starts at approximately 4 mm per voxel, decreasing by a factor of two at each subsequent level, and terminating at the input voxel size. These settings are commonly used in widely adopted registration packages such as ANTs (Avants et al., 2008) and NiftyReg (Modat et al., 2010). The standard deviations of the kernels used to smooth the final warp field and its gradient were based on settings reported in the FireANTs publication (Jena et al., 2024, which also shows that the registration is relatively insensitive to these values), supplemented by qualitative assessments on an internal dataset (Fischl et al., 2002). Similarly, the relative weight of the Dice component in the objective function was determined using qualitative results from the same internal dataset.

#### 2.2.6 Optimizing the Gaussian parameters ${\left\{\mu_{\text{k}},\sigma_{\text{k}}\right\}}_{\text{k}=1}^{\text{K}}$ and obtaining the final segmentation

Given the estimated bias field and atlas deformation parameters, the means and variances of the 16 (grouped) tissue classes are estimated with the EM algorithm; we note that it is EM and not GEM because the fixed bias field enables global maximization of the lower bound at each iteration. The EM procedure is carried out at a predefined resolution, to which both the input image and the probabilistic atlas are resampled. In our implementation, we set this resolution to 0.4 mm for *in vivo* scans, which balances anatomical detail and computational efficiency. For *ex vivo* scans, we set it to 0.2 mm (the resolution of the atlas) or the resolution of the scan, whichever is lower.

Since the EM algorithm estimates parameters at the level of grouped ROIs, the posterior responsibilities obtained in the final E-step provide a probabilistic segmentation into 16 tissue classes rather than into individual, fine-grained ROIs. Probabilistic estimates for the full set of ROIs are subsequently derived by redistributing the posterior probability mass of each tissue class across its constituent ROIs. This redistribution is performed according to the proportions given the atlas at each voxel (Puonti et al., 2016).

#### 2.2.7 Other simplifications and practical details

To further improve the efficiency and practicality of our method, we implemented some additional algorithmic and engineering simplifications:

- As mentioned above, we segment images at 0.4 mm isotropic resolution, instead of the 0.3 mm resolution used in the original NextBrain implementation. This reduces computational and memory demands by a factor of 2.4 while preserving anatomical detail.
- The entire pipeline, including FireANTs, is implemented in PyTorch, enabling seamless deployment on both GPU and CPU. PyTorch also facilitates efficient multi-threaded CPU execution.
- To accelerate atlas deformation, we store the probabilistic atlas as a set of sparse vectors defined within tight bounding boxes for each ROI, which allows rapid deformation of only the relevant subregions.
- In practice, GPU memory usage is dominated by the estimation of likelihood parameters, whereas actual computation is dominated by the atlas registration. When processing high-resolution *ex vivo* images at resolutions finer than 0.4 mm, we provide the option to offload likelihood optimization to the CPU (where memory is typically more abundant) while keeping the registration step on the GPU. Further details on memory are provided in Section 4 below.
- We removed 70 ROIs that showed poor registration quality across the five cases used to construct the atlas, all with very small volumes (<10 mm^3^). Voxels originally assigned to the removed ROIs were inpainted using the label of the nearest voxel outside this set. This procedure yields reduced run time and memory usage, and also simplifies label matching with the gold standard labels in the *ex vivo* MRI experiment in Section 3 (Casamitjana et al., 2025). The final model includes 264 anatomical regions; the list (including the list of removed labels) is provided in Supplementary Table S3.
- Processing one hemisphere at the time (required by the single-hemispheric nature of the atlas) also contributes to memory efficiency. Cross-hemispheric consistency is implicitly handled by the whole-brain segmentation from BrainFM, which guides and constrains the NextBrain segmentation.

## 3 Experiments and Results

### 3.1 Data

We use five different datasets to evaluate the proposed method, summarized in Table 1:

**Table 1.** Summary of datasets used for evaluation.

Dataset	N	Resolution	Mean age (years)	Labels
Ex vivo	21	0.2 mm	65	98 manual ROIs (gold standard) for one case (single hemi)
OpenBHB (in vivo)	3,227	1 mm	25.2	36 automated ROIs (silver standard) for all cases
MIRIAD (in vivo)	69	1 mm	69.5	185 repeated scans (test-retest)
HiP-CT (ex vivo)	6	0.3 mm	72	None
Visible Human (photographic)	1	0.15 mm	66	None

#### 3.1.1 *Ex vivo* MRI

This dataset consists of 21 specimens imaged with a Siemens 7 Tesla scanner at 0.12 mm isotropic resolution. The average age of the cohort is 65 years and the samples are from donors without neurological disorders. The samples comprise 12 single left hemispheres, 7 single right hemispheres, and two full brains. One of the full brains has a gold-standard segmentation: it corresponds to the case described in Edlow et al. (2019), for which we manually annotated 98 ROIs (including subcortical regions) in the right hemisphere at 0.2 mm isotropic resolution; these labels were released as part of the NextBrain dataset (Casamitjana et al., 2025). No gold-standard segmentations are available for the rest of the cases.

#### 3.1.2 *In vivo* MRI (OpenBHB)

OpenBHB (Dufumier et al., 2022) is a public meta-dataset with ∼1 mm isotropic T1-weighted volumes of 3,227 healthy subjects scanned at over 60 sites. The age range of the subjects is 6–86 years (mean: 25.2 years). No gold-standard segmentations are available for these scans. Instead, we obtained a silver standard by automatically segmenting these scans with the “supervised baseline” U-Net from Billot et al. (2023). This network was trained 1 mm isotropic T1-weighted scans to segment 35 ROIs using the FreeSurfer protocol. Despite the automated nature of the segmentation and the limited number of labels, this strategy enables evaluation over a much larger and more diverse sample than the *ex vivo* dataset.

#### 3.1.3 *In vivo* MRI (MIRIAD)

The Minimal Interval Resonance Imaging in Alzheimer’s Disease (MIRIAD) (Malone et al., 2013) is a public dataset with ∼1 mm isotropic T1-weighted volumes of 69 subjects (46 mild–moderate Alzheimer’s subjects and 23 controls, age at entry 69.5 ± 7.1 years, 31 men). It comprises 708 scans acquired with the same scanner and pulse sequences at intervals of 2, 6, 14, 26, 38 and 52 weeks, 18 and 24 months from baseline, with up to 12 scans per individual. No gold-standard segmentations are available for this dataset but, because 185 of the scans are repeat acquisitions of the same timepoints, they can be used to assess test-retest reliability.

#### 3.1.4 *Ex vivo* hierarchical phase-contrast tomography (HiP-CT)

HiP-CT is an advanced X-ray imaging technique used to visualize intact human organs *ex vivo* at multiple scales, from whole-organ structure down to cellular detail (Walsh et al., 2021). It leverages phase contrast generated by X-ray wavefront distortions using a synchrotron source to achieve resolutions as high as 1 μm. Walsh et al. (2021) have made 6 brain HiP-CT scans available (4 males, 2 females, ages 63–80). The scans were downloaded from the Human Organ Atlas (HOA) repository at a resolution that was closest to 0.3 mm isotropic, which resulted in a range of resolutions spanning from 0.2 to 0.3 mm. We excluded case LADAF-2020-31, as it was among the earlier acquisitions and its image quality was notably inferior to that of the other cases. No gold- or silver-standard segmentations are available for these scans.

#### 3.1.5 *Ex vivo* visible human 2.0

This is a high-resolution anatomical dataset of the head and neck of a 66-year-old male donor, focused on axial cryosection photography acquired every 0.15 mm (Ratiu et al., 2003). Tissue contrast was optimized to distinguish fine brain structures. We normalized the photographs by median intensity to correct for illumination inhomogeneity across slices. No gold- or silver-standard segmentation is available for this dataset.

### 3.2 Results

#### 3.2.1 Segmentation of ultra-high resolution *ex vivo* MRI

**Fig. 3.**
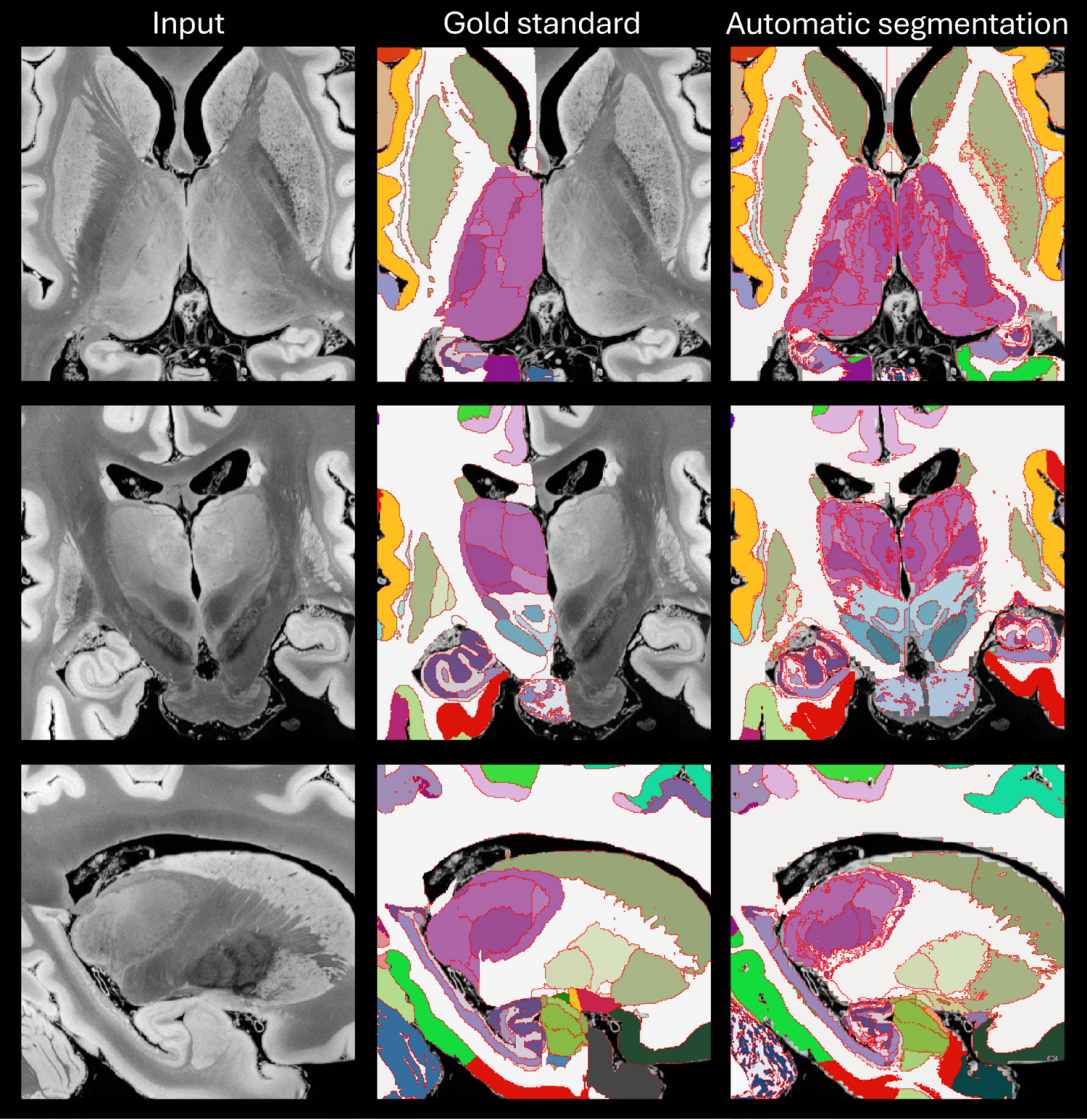
Axial (top row), coronal (middle), and sagittal slices (bottom) of the publicly available *ex vivo* scan from Edlow et al. (2019), along with the gold standard (manual delineations, only right hemisphere available) and the automated segmentation with NextBrain. We note that the input was resampled from 0.1 mm to 0.2 mm resolution, which is the voxel size of the manual segmentation, and also the native resolution of NextBrain. We also note that the manual delineation is less granular than the automated segmentation (98 ROIs vs 264).

Figure 3 presents segmented slices from the case described in Edlow et al. (2019), including both the manually labeled gold standard and the corresponding automated segmentations. Despite the difference in granularity between the two segmentations (98 manually defined ROIs versus 264 in the automated output), the overall agreement between them is remarkably strong. Table S2 in the Supplementary Material summarizes the Dice scores for the 98 manually labeled ROIs, obtained by clustering the automated segmentation to align with the gold standard definitions. ROI sizes are also included in the table. As expected, there is a clear association between region size and Dice score. Larger structures (e.g., cerebral and cerebellar white matter and cortex) achieve Dice values near 0.9. Smaller regions tend to show lower scores, although only a handful fall below 0.4, which remains adequate for localization purposes. Overall, the Dice performance (0.610 ± 0.207) closely matches that of the original method (0.626 ± 0.180), indicating comparable segmentation accuracy.

**Fig. 4.**
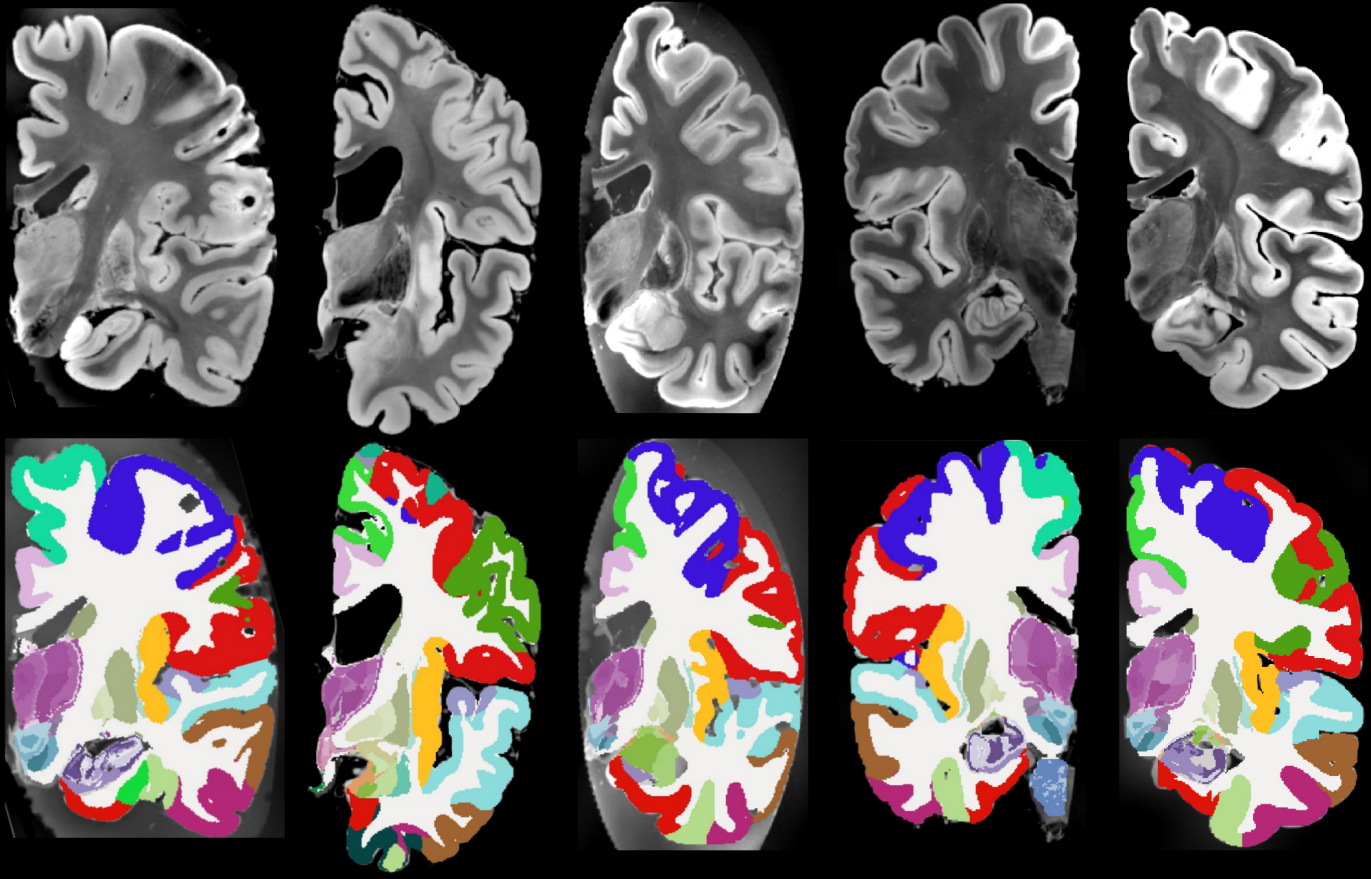
Five example subjects from the ex-vivo MRI data set. Coronal view of the MRI data (top) and the corresponding automated segmentation using NextBrain (bottom).

Qualitative results are shown in Figure 4, which displays sample coronal segmentations of five other subjects from the *ex vivo* MRI data set. As no reference segmentations are available for 20 out of the 21 subjects, we instead report the volumes of the substructures of the hippocampus, thalamus and amygdala in Tables 2, 3, and 4, and compare them with those from other existing *ex vivo* atlases (Iglesias et al., 2015, 2017, 2018). Because these atlases were constructed from manual delineations based on anatomical protocols that differ from those of NextBrain, we mapped each label in the atlases to the closest corresponding NextBrain ROI or set of ROIs. The volumes and ROI correspondences are listed in Tables 2, 3, and 4. In general, the volumes agree quite well, with two exceptions: small or narrow structures like the granular layer of the dentate gyrus in the hippocampus; and the posterior regions of the thalamus, due to discrepacies in manual delineation protocols—in Iglesias et al. (2018), the boundary between mediodorsal and pulvinar regions is more anterior than in NextBrain, and the whole thalamus is also generally larger (6,540 vs 5,970 mm^3^).

**Table 2.** Average volumes of the hippocampal subfields from Iglesias et al. (2015) (N = 15) and from the automated NextBrain segmentations on the *ex vivo* MRI data (N = 20).

Hippocampus				
Structure	(Iglesias et al., 2015)			Automated NextBrain segmentations
	Combined structures	Volume μ (mm³)	Combined structures	Volume μ±σ (mm³)
Subiculum	Pre/Post-subiculum Subiculum	624	Caudal/uncal/rostral-subiculum	846.72±109.67
Alveus	Alveus	320	Alveus	427.61±94.01
CA1	CA1	520	Stratum-radiatum-caudal/uncal/rostral/-CA1 Stratum-pyramidale-caudal/uncal/rostral-CA1 Stratum-oriens-caudal/uncal/rostral-CA1	873.71±124.55
CA2/3	CA2/3	179	Stratum-radiatum-caudal/uncal/rostral-CA2 Stratum-pyramidale-caudal/uncal/rostral-CA2/CA3 Stratum-oriens-caudal/uncal/rostral-CA2/CA3	239.48±45.42
CA 4	CA 4	211	pyramidal-cells-caudal/uncal/rostral-CA4 molecular-layer-caudal/uncal/rostral-dentate-gyrus	348.99±73.72
Granular layer dentate gyrus	GC–DG	244	granular-layer-caudal/uncal/rostral-dentate-gyrus	54.27±10.94
Hippocampal Amygdala transition area	HATA	50	amygdalohippocampal-transition-area	14.90±2.96
Fimbria	Fimbria	92	Fimbria	51.91±22.48
Molecular layer	Molecular layer	466	molecular-layer-caudal/uncal/rostral-dentate-gyrus stratum-moleculare-caudal/uncal/rostral-CA1/CA2/CA3	714.08±135.89

**Table 3.** Average volumes of nuclei of the amygdala from Iglesias et al. (2017) (N = 10) and from the automated NextBrain segmentations on the *ex vivo* MRI data (N = 20).

Amygdala				
Structure	(Iglesias et al., 2017)		Automated NextBrain segmentations	
	Combined structures	Volume μ±se (mm³)	Combined structures	Volume μ±σ (mm³)
Lateral nucleus	Lateral nucleus	453±31.4	lateral-nucleus	393.69±40.78
Basal nucleus	Basal nucleus	300.9±19.2	basolateral-nucleus (basal-nucleus)	447.48±53.29
Central nucleus	Central nucleus	32.5±7	medial/lateral-subdivision-central-nucleus	37.51±10.22
Medial nucleus	Medial nucleus	21.8±5.6	rostral-subdivision-medial-nucleus	39.87±12.41
Cortico-amygdaloid Transition Area	CAT	174.8±17.3	amygdalocortical-(corticoamygdaloid) transition-area	152.18±25.67
Paralaminar nucleus	Paralaminar nucleus	31.9±6.4	paralaminar-nucleus	13.16±3.49
Anterior Amygdala Area	Anterior Amygdala Area	39.8±7.9	anterior-amygdaloid-area	113±20.75

**Table 4.** Average volumes of nuclei of the thalamus from Iglesias et al. (2018) (N = 6) and from the automated NextBrain segmentations on the *ex vivo* MRI data (N = 20).

Thalamus				
Structure	(Iglesias et al., 2018)		Automated NextBrain segmentations	
	Combined structures	Volume μ (mm³)	Combined structures	Volume μ±σ (mm³)
Anterior	Anterovetral	188.74	anteroventral/medial/dorsal-nucleus-thalamus	139.90±51.71
Lateral	Laterodorsal Lateral posterior	184.84	lateral/central-dorsal-nucleus-thalamus dorsal-division-central- lateral-nucleus	72.20±28.44
Ventral	Ventral anterior Ventral anterior magnocellular Ventral lateral anterior Ventral lateral posterior Ventral posterolateral Ventromedial	2393.13	parvo/magnocellular-division-va ventral/dorsal/medial-subdivision-vlc rostral-division-vl basal-ventral-medial-nucleus rostral/caudal-division-ventral- posterior-lateral-nucleus ventral-posterior-inferior-nucleus ventral-medial-nucleus-thalamus ventral-posterior-medial-nucleus parvocellular-division-vpm	2244.03±527.99
Intralaminar	Central medial Central lateral Paracentral Centromedian Parafascicular	453.38	central-medial-nucleus-thalamus lateral-subdivision-central-nucleus paracentral/centromedian/ parafascicular/fasciculosus/ subparafascicular/rhomboid-nucleus-thalamus lateral/medial-division-centromedian-nucleus-thalamus periventricular-area-thalamus	375.21±114.89
Medial	Paratenial Reuniens (medial ventral) Mediodorsal medial magnocellular Mediodorsal lateral parvocellular	854.74	parataenial/reuniens/ intermediodorsal-nucleus-thalamus magnocellular/parvocellular/densocellular/ multiform-division-of-md	1266.78±200.19
Posterior	Lateral/Medial-Geniculate Limitans (suprageniculate) Pulvinar (anterior/medial/lateral/inferior)	2117.44	dorsal-medial/lateral-geniculate-nucleus suprageniculate/posterior-nucleus-thalamus lateral-posterior-nucleus-thalamus limitans/magnocellular-nucleus medial/lateral/inferior/anterior-nucleus-pulvinar pulvinar-thalamus superficial/diffuse-pulvinar-nucleus	1569.23±249.28
Reticular	Reticular	350.53	magno/parvocellular-division-reticular-nucleus reticular-nucleus-thalamus	358.11±85.53

#### 3.2.2 Segmentation of *in vivo* MRI

##### 3.2.2.1 Dice and volume correlation on OpenBHB

As with most human brain MRI segmentation tools, one of the primary applications of NextBrain is the analysis of *in vivo* scans acquired at near 1 mm isotropic voxel size. However, evaluating subregion-level Dice scores on such data would require manual annotations of structures that often cannot be reliably delineated at this resolution. Instead, we assess performance at the whole-ROI level by merging the automatically segmented subregions to approximate the FreeSurfer protocol (Fischl et al., 2002), and comparing against the silver-standard generated via automated segmentation. Even though using silver-standard labels is not ideal, the ability to evaluate on this heterogeneous dataset further strengthens the generalizability of the assessment.

**Fig. 5.**
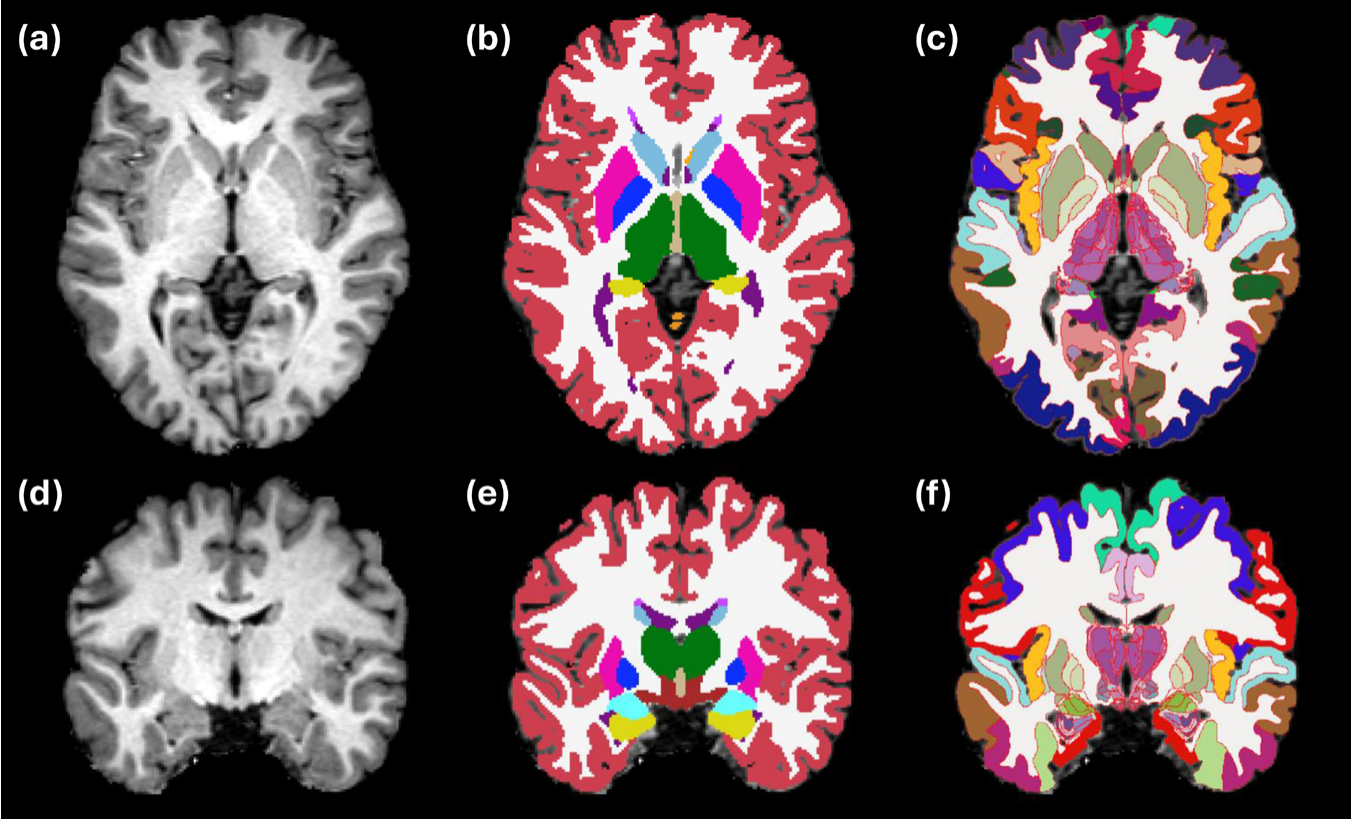
Segmentation of sample subject (368981431172) from OpenBHB dataset. (a) Axial slice of input. (b) Silver-standard segmentation with supervised U-Net for 1 mm T1 scans. (c) Segmentation using NextBrain, with ROI boundaries highlighted in red. (d,e,f) Coronal slice of the same case.

Figure 5 illustrates the segmentation results for a representative case from the OpenBHB dataset. Our method produces segmentations that are qualitatively very similar to those of the baseline, while also leveraging visible subregional boundaries and additional anatomical labels to more precisely align the fine-grained NextBrain labels with the underlying image data. Noticeable improvements include: clearer separation between the putamen and adjacent cortex; subdivision of the thalamus that reflects the darker contrast of medial and posterior nuclei; more accurate delineation of the lateral boundary of the thalamus; subdivision of the pallidum; and improved characterization of the hypothalamic and subthalamic regions, among others. Additional qualitative results on non-standard MRI contrast (R2*) acquired using ultra-high field MRI (Alkemade et al., 2020) are shown in Supplementary Figure S2.

Table 5 presents the quantitative results for both Dice scores and volume correlations. There is a strong correlation between ROI volumes computed by the two methods. Dice scores comparing the segmentations directly are also consistently high across all regions. These findings support the robustness of the segmentation approach at the whole-region level.

**Table 5.** The average Dice scores and volume correlations against the silver standard on the OpenBHB data set.

ROI	Dice	Correlation (95% C.I.)
Cerebral white matter	0.908	0.934 (0.929, 0.938)
Cerebral cortex	0.865	0.920 (0.915, 0.925)
Cerebellar white matter	0.837	0.861 (0.852, 0.870)
Cerebellar cortex	0.910	0.909 (0.903, 0.915)
Thalamus	0.914	0.944 (0.940, 0.948)
Caudate	0.894	0.816 (0.804, 0.827)
Putamen	0.927	0.960 (0.957, 0.963)
Pallidum	0.894	0.899 (0.892, 0.905)
Hippocampus	0.881	0.867 (0.858, 0.875)
Amygdala	0.832	0.813 (0.801, 0.824)

The NextBrain ROIs are grouped to match the coarser segmentation from the supervised U-Net.

**Fig. 6.**
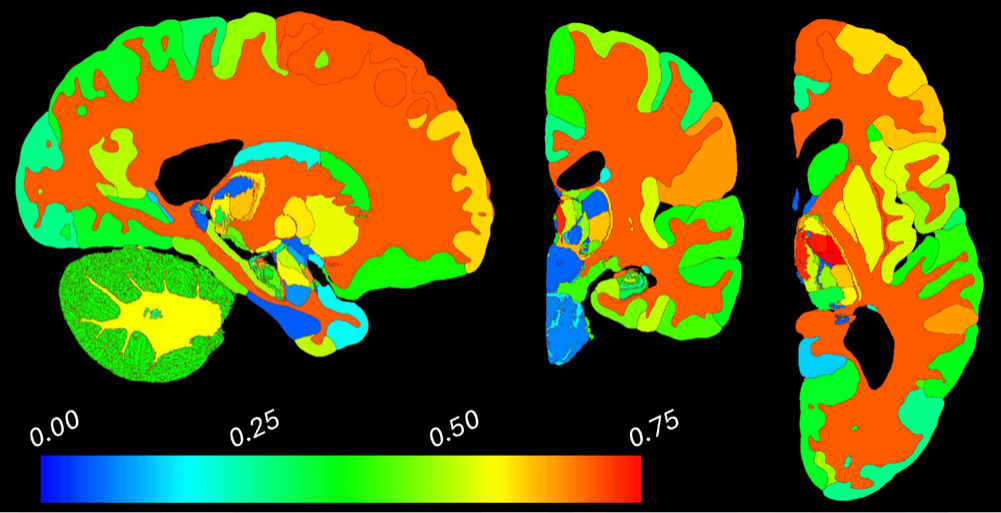
Slices of map of Spearman correlation for ROI volume vs age using OpenBHB dataset, restricted to subjects aged 35 years and over (n=431 subjects, mean age: 57.9 years); we chose this age range as 35 is approximately the age when age-related atrophy begins.

Figure 6 illustrates the ROI-wise correlation between age and volume (an “aging map”) based on our high-resolution parcellation. The results follows similar trends as those obtained with the classical Bayesian tool (figure 5b of Casamitjana et al., 2025), including stronger associations in the frontal cortex, medial regions of the thalamus, the anterior region of the caudate, the subicular areas of the hippocampus, or the insula. Some minor discrepancies arise, especially in smaller nuclei. These are mostly a consequence of the discrepancies in the atlas registration, due to the different objective functions (log-likelihood and membrane energy vs LNCC and dice with FireANTs).

##### 3.2.2.2 Test-retest reliability with MIRIAD

**Fig. 7.**
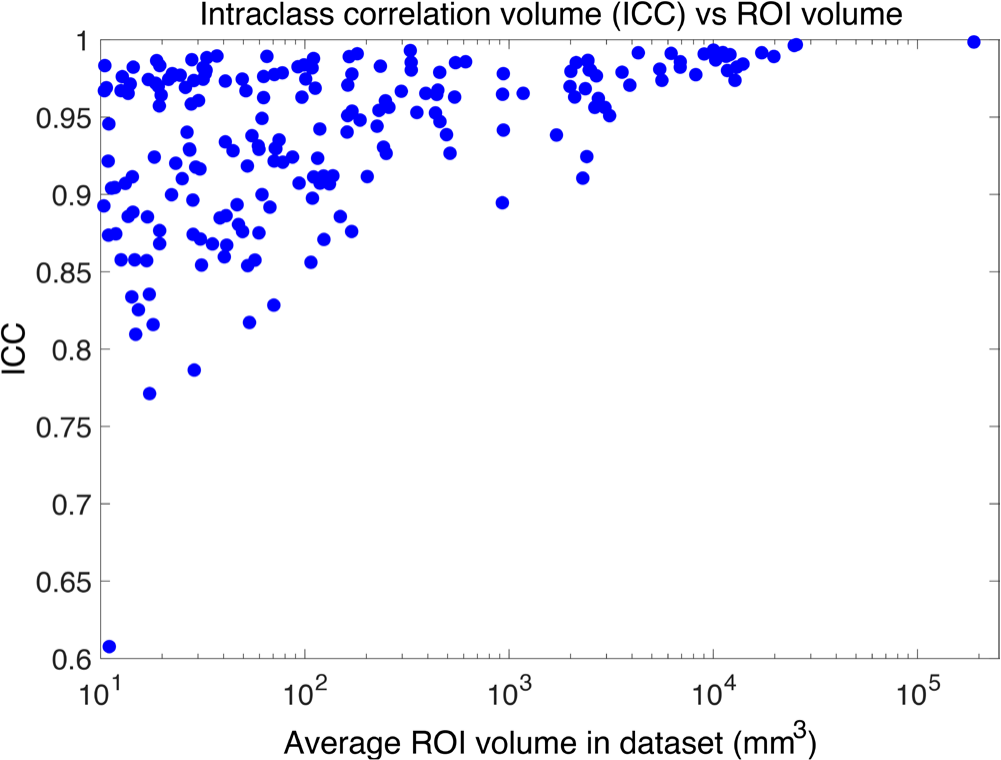
Scatter plot of ICC vs. average ROI volume in test-retest experiment on MIRIAD dataset.

When processing *in vivo* MRI scans at approximately 1 mm resolution, NextBrain necessarily relies more heavily on geometric priors encoded in the atlas to delineate many internal boundaries—some of which are more reliably inferred than others from the outer contours of brain regions. It is, therefore, important to evaluate the test-retest reliability of the method. For this purpose, we used the MIRIAD dataset: despite not having gold-standard labels, it has 185 timepoints with repeat acquisitions. We computed the intra-class correlation coefficient (ICC) for the volumes of all 264 ROIs, averaged across the left and right hemispheres. An complete list is provided in Table S3 of the Supplementary Material. The vast majority of ROIs exhibit excellent reliability, with ICC values exceeding 0.8. Notably, all ROIs with lower ICCs are very small in volume (Fig. 7): all with ICC <0.9 have a volume below 170 mm^3^ (except the myelencephalon), and all with ICC <0.8 fall below 30 mm^3^.

#### 3.2.3 Segmentation of unconventional, high-resolution *ex vivo* modalities

**Fig. 8.**
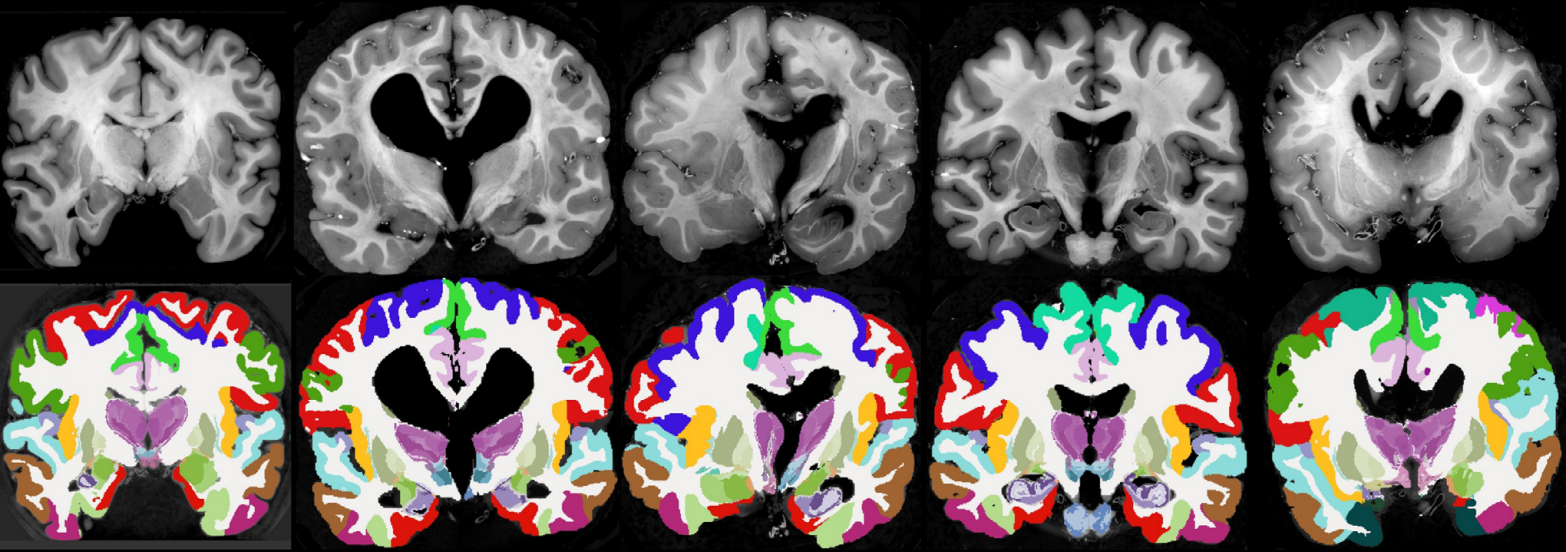
Coronal slices of the five HiP-CT scans (top) and their corresponding automated segmentations using NextBrain (bottom).

**Fig. 9.**
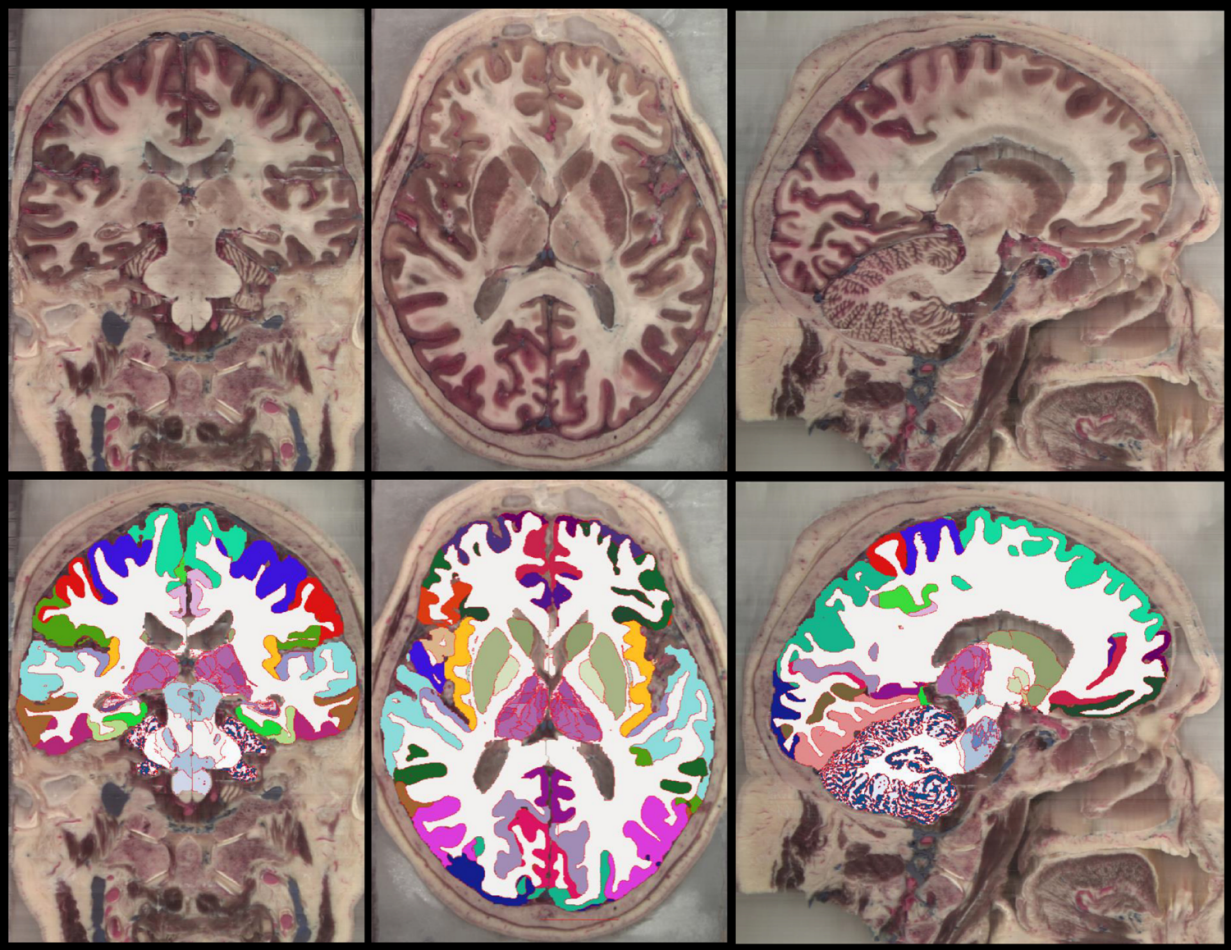
Coronal (left), axial (middle), and sagittal slices (left) of the Visible Human 2.0 dataset, along with automated segmentation computed with NextBrain.

Figure 8 shows coronal slices of all five HiP-CT scans along with the segmentation produced by NextBrain. Notably, our method can tackle significant variations from normal appearing anatomy as exemplified by the three subjects with substantially enlarged or deformed ventricles. Figure 9 shows orthogonal slices from the Visible Human dataset alongside the corresponding segmentation. Given the higher native resolution of this dataset compared to NextBrain, the segmentation was performed at half resolution (0.3 mm isotropic) to reduce computational load. The segmentations were computed on grayscale images; although our method naturally extends to RGB inputs (similar to Bayesian segmentation, with mean vector and covariance matrices replacing scalar means and variances), processing full color volumes at this resolution would require prohibitive amounts of memory. The high-resolution and excellent contrast of both *ex vivo* data sets allow for accurate delineation of complex structures such as the thalamic nuclei. The Visible Human data has increased detail in regions that are often difficult to visualize in MRI, for example, the dentate gyrus of the cerebellum, as seen in the sagittal slice.

#### 3.2.4 Run times

To reflect typical research settings, we evaluated the software on two hardware configurations that are representative of research environments:

1. Modern: Intel Xeon w9-3475X CPU (36 cores, 72 threads), NVIDIA RTX 6000 GPU. These were released in 2023 and 2020, respectively.
2. Legacy: Intel Xeon Gold 6226R CPU (32 cores, 64 threads), NVIDIA Quadro RTX 8000 GPU. These were released in 2020 and 2018, respectively.

Table 6 shows the average run times and peak memory usages over 20 random cases of the OpenBHB dataset, using default resolution settings. On the modern hardware, a case processes in approximately 5 minutes on a GPU, or 22 minutes on the CPU. Run times remain practical on the legacy workstation: 9 minutes on GPU, and 34 minutes on CPU. The memory footprint is approximately ∼10 GB on the CPU and ∼20 GB on the GPU; the latter is imposed by the neural network in BrainFM. If the GPU is not used, the peak CPU memory usage is the 20 GB required by the neural network. While these requirements may be considered demanding for consumer desktops, they are fairly standard within research computing environments.

**Table 6.** Peak memory usage and run times for GPU and CPU versions on two different workstations (modern and legacy; see details in main text).

GPU memory	CPU memory	Time (modern/GPU)	Time (legacy/GPU)	Time (modern/CPU)	Time (legacy/CPU)
19.5 ±2.9 GB	10.7 ±1.6 GB	4.7 ±0.4 minutes	8.9 ± 0.5 minutes	22.0 ±2.1 minutes	33.8 ±2.2 minutes

We note that, when running solely on the CPU, the total memory usage is equal to that listed under GPU, that is, ∼20 GB.

## 4 Software Toolbox

The code and necessary files to run it are distributed as a part of the FreeSurfer software package. Once FreeSurfer is installed and configured, and the atlas files have been fetched (the code prompts the user to download them at its first use), the code can be executed by calling:

mri_histo_atlas_segment_fireants --i <scan> --o <out_dir> --device <dev> --side <side> --mode <mode>

The algorithm is optimized to balance speed and memory to strike a balance between execution times and the required resources. For example, the individual structure locations in the high-resolution atlas are stored as bounding boxes and the atlas is resampled only within the bounding box when computing the final probabilities to save memory. Similarly many of the atlas operations are done one structure (channel) at a time, which is slightly slower but more memory efficient. Combined with optimization of the BrainFM network inference, the segmentation now requires only 10 GB of CPU memory and 20 GB of GPU memory as shown in Table 6.

The list of all possible input arguments and their descriptions are shown in Table 7 and summarized here. I/O flags aside, the remaining options can be roughly divided into two categories: *(i)* controlling memory and speed, and *(ii)* controlling the nonlinear registration computed with FireANTs. The options in the first category include --device and --device_registration, which allow the user to choose between the CPU and the GPU, or to offload the memory heavy registration part to the CPU if working with a GPU with limited memory. The --skip flag can be used to perform integer downsampling of the atlas and the input image to save memory when estimating the GMM parameters in the EM step, at the expense of slightly less accurate parameter estimation (and thus segmentation). The number of CPU threads can be controlled with the --threads flag (by default, all available threads are used).

**Table 7.** The description and default values of all possible NextBrain command line arguments.

Flag	Type	Default value	Description
--i	string	None	Path to input image (required).
--o	string	None	Output path (required).
--mode	string	None	Type of input (required). Options: invivo, exvivo, cerebrum, hemi
--side	string	None	Hemisphere to segment (required). Options: left, right.
--bf_mode	string	dct	Bias field basis function model. Options: dct, polynomial, hybrid
--write_rgb	binary	false	If flag is present, save an RGB image based on the posterior probabilities.
--write_bias_corrected	binary	false	If flag is present, save bias corrected input image.
--device	string	cpu	Device to use. Options: cpu, cuda.
--device_registration	string	Same as --device	Use different device for registration. Options: cpu, cuda.
--threads	integer	−1	Number of threads to use. Default uses all available.
--skip	integer	1	Downsampling factor for estimating EM parameters.
--resolution	float	0.4	Resolution of the output segmentation
--smoothing_steps_HRmask	integer	3	Number of smoothing steps for upsampling the 1 mm segmentation mask.
--skip_bf	binary	false	If flag is present, skip bias field correction.
--smooth_grad_sigma	float	1.0	Gradient smoothing parameter (FireAnts registration).
--smooth_warp_sigma	float	0.25	Warp smoothing parameter (FireAnts registration).
--optimizer_lr	float	0.5	Optimizer step size (FireAnts registration).
--cc_kernel_size	int	7	Cross-correlation window size (FireAnts registration).
--rel_weight_labeldiff	float	2.5	Relative weight of labels (FireAnts registration).
--save_atlas_nonlinear_reg	binary	false	If flag is present save the nonlinearly registered atlas.
--save_field	binary	false	If flag is present save the nonlinear deformation field.
--save_jacobian	binary	false	If flag is present save the Jacobian determinant (log10).
—yaml_path	string	None	path of custom YAML files to define groups of ROIs.

The options in the second category include the --smooth_grad_sigma and --smooth_warp_sigma flags, which control the smoothness of the gradient and warp fields, --optimizer_lr, which is the learning rate for the Adam optimizer, --cc_kernel_size, which controls the window size for computing the cross-correlation metric, and --rel_weight_labeldiff, which can be used to balance the cross-correlation and Dice loss objectives. Additionally, there are a number of flags (--save_*) that enable the user to save the nonlinearly registered atlas, the nonlinear deformation field, and the Jacobian determinant of the deformation field, for downstream analyses or for assessing the quality or smoothness of the atlas registration.

The resolution of the output segmentation is $0.4{\text{ mm}}^{3}$ by default as our previous experience with *ex vivo* atlases (Iglesias et al., 2015, 2017, 2018) has shown that using a higher resolution than that of the input scan enables the method to produce segmentations without spatial aliasing. However, the output resolution can be controlled using the --resolution flag.

The software uses a required argument (--mode) to specify the type of input, which can be a standard *in vivo* scan, an *ex vivo* scan, a single hemisphere, or only the cerebrum (without cerebellum and brain stem). The selected mode determines which labels are included into the modeling, and is also propagated to BrainFM.

In order to make the algorithm more flexible, and for tuning it for specific image contrast and structures, the users can create their own structure groupings for estimating the GMM parameters and controlling how the synthetic image is generated from the atlas. The structure groupings for the GMM are controlled by two YAML files: gmm_components_fireants.yaml and combined_atlas_labels_fireants.yaml, which by default encode our prior knowledge for optimal structure groupings for MRI segmentation. As an example, let us assume that the user has a scan where the internal segment of globus pallidus (label 206) is very visible in the scan. To model this structure separately, the user would first create a new class called, for example, Internal Segment Pallidum, in the combined_atlas_labels_fireants.yaml file, and list label 206 under this structure (while removing it from the pallidum class). Next, the user would add the class, with exactly the same name, to the gmm_components_fireants.yaml and decide how many Gaussian distributions should be used to model its intensities. To make the non-linear registration aware of the contrast, the user would add the structure, with exactly the same name, to a file called recipe_intensities_cheating_image_fireants.yaml, and decide how its intensity should be generated from the seven structures than can be always reliably segmentation using BrainFM (see Supplementary Table S1 for an example). The custom YAML files are placed in a directory chosen by the user, which is passed to the code via the flag --yaml_path.

Finally, we note that extracerebral tissue (including CSF) must be masked out as part of preprocessing, since it is not modeled by NextBrain. For this purpose, we use the BrainFM segmentation, which is automatically resampled to the voxel space of the input scan. Nearest neighbor interpolation would lead to jagged boundaries when analyzing high-resolution scans (e.g., *ex vivo* data), since the native resolution of BrainFM outputs is 1 mm isotropic. Instead, we use a small pipeline comprising linear interpolation of the binary mask, smoothing, and thresholding at the 0.5 level; the user has control over the smoothing via the --smoothing_steps_HRmask parameter (default: 3 steps). Alternatively, one could consider using segmentation methods that operate natively at higher resolutions. However, BrainFM is—to the best of our knowledge—the only publicly available method that can handle presence or absence of a whole hemisphere, cerebellum, and/or brainstem. Other methods would preclude the analysis of *ex vivo* scans with these structures missing.

We further note that the choice for the mask smoothing parameter parameter represents a compromise between smoothness and accuracy. Poor masking can lead to biased volume estimates for ROIs that share a boundary with the extracerebral regions (typically CSF). While the relative error may be low for large ROIs like the white matter, it may be non-negligible for smaller ROIs neighboring the CSF, for example, the mediodorsal nucleus of the thalamus.

## 5 Discussion and Conclusion

In this work, we introduced a fast segmentation method based on the NextBrain histological atlas, capable of accurately parcellating a large number of brain ROIs across several modalities. A key strength of the proposed approach is its computational efficiency, which enables practical application to high-resolution datasets without incurring prohibitive computational costs. For example, segmenting the Visible Human volume at 0.3 mm isotropic resolution required only ∼90 minutes on a standard eight-core workstation, compared to nearly a week using the original implementation. For *in vivo* scans, our method completes segmentation in approximately 20 minutes on the same hardware, whereas the original code required 2–3 days. When a GPU with sufficient memory (∼24 GB) is available, segmentation time can be reduced to under 5 minutes.

Quantitative validation across several benchmarks, including manual annotations, volume comparisons, and comparison against established segmentation methods, demonstrated that our approach achieves competitive accuracy. As expected, segmentation performance is positively correlated with ROI size, but even small regions such as thalamic subregions, hippocampal subfields, and subregions of the amygdala were reliably identified. Moreover, the method captures biologically meaningful variation: age-volume correlations derived from the segmentations revealed region-specific patterns consistent with previous literature, supporting both the anatomical fidelity and practical relevance of the results.

Our software ships with a default set of parameters that encode our prior experience and knowledge on MRI, Bayesian segmentation, and the NextBrain atlas, and which yield robust results across a wide spectrum of resolution and contrast (even photographic). At the same time, the software is highly configurable, such that an advanced user can tweak these parameters to exploit highly specific intensity characteristics of a specialized acquisition, for example, an MRI pulse sequence capable of highlighting some determined brain nuclei. We also note that, even though our software can handle images of very different contrasts and resolutions (as shown in the results), it is not strictly contrast-agnostic, since there are some design choices that are contrast dependent. Nevertheless, we have tried to minimize their impact, by making them a function of tissue types rather than absolute intensities (e.g., the heuristic intensity estimates for the synthetic MRI described in the Supplementary Material).

Three main limitations should be acknowledged. First, our method inherits the constraints of the NextBrain atlas, which was derived from only five cases of older, healthy individuals. Albeit mitigated by the BrainFM segmentation (trained on a much larger and diverse population), generalization to broader or clinical populations may be limited. Second, although our use of synthetic anatomical volumes substantially accelerates registration, it may not always achieve the accuracy of probabilistic approaches employed in the original Bayesian algorithm, for example, in convoluted regions where the probabilistic labels are blurry and yield averaged intensities that may not faithfully represent the underlying tissue; this is compounded with differences in the regularizer and leads to slightly different results, for example, in Figure 6. And third: the NextBrain-specific label set used in this work limits direct comparisons with alternative segmentation methods or classical pipelines. Consequently, the reported accuracy should be interpreted as absolute performance on this particular label scheme, rather than as a measure directly comparable across methods.

Overall, our new method offers a powerful and accessible tool for fast, high-resolution brain segmentation. By combining detailed histological priors with a lightweight implementation, it bridges the gap between expert-curated atlases and real-world neuroimaging needs. Its speed, simplicity, and robustness make it especially well-suited for large-scale studies at the subregion level. Crucially, by removing the need for specialized software or manual anatomical expertise, this freely available tool democratizes access to fine-grained brain segmentation, enabling a wider community of researchers to explore the brain at unprecedented spatial detail.

## Supplementary Material

Supplementary Material

## Data Availability

A ready-to-use tool is available as part of FreeSurfer: https://surfer.nmr.mgh.harvard.edu/fswiki/HistoAtlasSegmentation. As with the rest of FreeSurfer, the source code is available on GitHub: https://github.com/freesurfer/freesurfer/tree/dev/mri_histo_util. The Hip-CT scans can be accessed using the following digital object identifiers (DOIs): Subject LADAF-2021-17: http://doi.org/10.15151/ESRF-DC-1773964937. Subject S-20-29: http://doi.org/10.15151/ESRF-DC-1773961413. Subject S-21-33: http://doi.org/10.15151/ESRF-DC-2222698134. Subject S-21-46: http://doi.org/10.15151/ESRF-DC-2222698126. Subject S-22-16: http://doi.org/10.15151/ESRF-DC-2222698142.
