# Supplementary material for: Fast segmentation with the NextBrain histological atlas
Source: Imaging Neurosci (Camb). 2026 May 26;4:IMAG.a.1244. doi: 10.1162/IMAG.a.1244 (PMC13214569; doi:10.1162/IMAG.a.1244)
Supplement: Supplementary Material [file IMAG.a.1244_supp.pdf]

Supplementary Material

Heuristic intensity estimates for creating a synthetic MRI scan

When building the Gaussian synthetic anatomical volume, the mean intensities of the different tissue types (16, by default) are computed as linear combinations of seven classes than can always be reliable estimated from the target scan using the BrainFM segmentations: white matter (μ^{wm}), gray matter (μ^{gm}), cerebellar white matter (μ^{cwm}), cerebellar gray matter (μ^{cgm}), caudate (μ^{ca}), putamen (μ^{pu}), and pallidum (μ^{pa}). The default combinations are described below; the user can modify the configuration files to specify their own, if they want to exploit prior knowledge on the intensities of the scan to segment. As explained in Section 2.2, the means do not have to be very precise, thanks to the robustness of the objective function in the registration.

With the default settings, the seven tissue types that directly correspond to the seven classes are assigned their respective means. For the other nine tissue types, we first compute the midpoint between the gray and white matter $\mu^{\text{mid}} = 0.5(\mu^{\text{wm}} + \mu^{\text{gm}})$ and a fraction $\delta = (\mu^{\text{wm}} - \mu^{\text{gm}})/16$. Using these values, we create the following entries:

$$\mu^{\text{th-lat}} = \mu^{\text{mid}} + 2\delta$$ Lateral part of the thalamus

$$\mu^{\text{th-med}} = \mu^{\text{mid}} - 2\delta$$ Medial part of the thalamus

$$\mu^{\text{rn}} = \mu^{\text{wm}} + 9\delta$$ Red nucleus

$$\mu^{\text{gm-bs}} = \mu^{\text{wm}} - \delta$$ Compact brainstem white matter

$$\mu^{\text{wm-bs}} = \mu^{\text{wm}} + 6\delta$$ Diffuse brainstem white matter

$$\mu^{\text{hypo}} = \mu^{\text{mid}} - 3\delta$$ Hypothalamus

$$\mu^{\text{ma-body}} = \mu^{\text{wm}}$$ Mamillary bodies

$$\mu^{\text{dn-cb}} = \mu^{\text{mid}} - \delta$$ Dentate nucleus

$$\mu^{\text{wm-hippo}} = \mu^{\text{mid}} + \delta$$ White matter of the hippocampus

These entries yield the final linear combinations, found in the default configuration file and in Table S1.

Table S1: Default linear weights for creating synthetic MRI scans.

Tissue type	μ^{wm}	μ^{gm}	μ^{cwm}	μ^{cgm}	μ^{ca}	μ^{pu}	μ^{pa}
Cerebral white matter	1.0	0	0	0	0	0	0
Cerebral gray matter	0	1.0	0	0	0	0	0
Cerebellar white matter	0	0	1.0	0	0	0	0
Cerebellar cortex	0	0	0	1.0	0	0	0
Caudate	0	0	0	0	1.0	0	0
Putamen	0	0	0	0	0	1.0	0
Lateral thalamus	0.625	0.375	0	0	0	0	0
Medial Thalamus	0.375	0.625	0	0	0	0	0
Pallidum	0	0	0	0	0	0	1.0
Red nucleus	1.5625	-0.5625	0	0	0	0	0
Compact brainstem white matter	0.9375	0.0625	0	0	0	0	0
Diffuse brainstem white matter	1.375	-0.375	0	0	0	0	0
Hypothalamus	0.3125	0.6875	0	0	0	0	0
Mammillary bodies	1.0	0	0	0	0	0	0
Dentate nucleus of the cerebellum	0.4375	0.5625	0	0	0	0	0
Hippocampal white matter	0.5625	0.4375	0	0	0	0	0

Qualitative comparison of atlas deformation with FireANTs and Casamitjana et al. (2025)

Figure S1 shows the Jacobian determinants of the deformation fields mapping the test subject distributed with FreeSurfer ("bert") to the NextBrain atlas, using the classical Bayesian approach (Casamitjana et al., 2025) and the method proposed in this article (both with default settings). Both methods have several positive clusters (i.e., expansion of coordinates, meaning compression of the atlas) around the different ventricles and cortical sulci, reflecting the small volume of CSF in bert. There are also similar trends for the the negative clusters. With these default settings, the fields obtained with our new approach are generally less regularized, but the user can adjust this behavior with the options `--smooth_grad_sigma` and `--smooth_warp_sigma` (see Section 4).

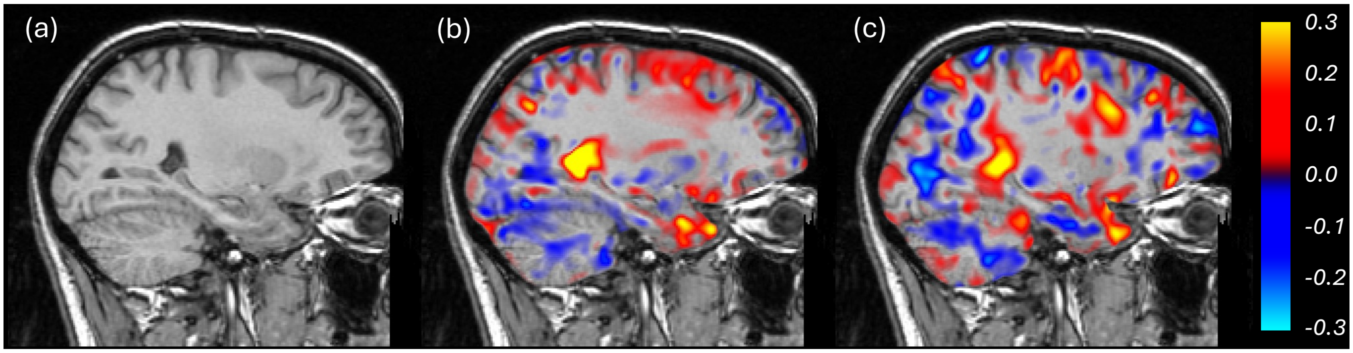

Figure S1: (a) Sagittal slice of "bert", the test subject distributed with FreeSurfer. (b) Same slice, with Jacobian determinant of atlas deformation from Casamitjana et al. (2025) overlaid (in base-10 logarithmic scale). (c) Same as (b), but with the method proposed in this article, based on FireANTs. Both methods were run with default settings.

Ex vivo comparison against the gold standard (manual labeling) from (Edlow et al., 2019)

Table S2: Volumes and Dice scores across all ROIs on the *ex vivo* sample from Edlow et al. (2019). For comparison, we also provide the Dice scores obtained with the classical Bayesian approach (reported in Casamitjana et al. 2025).

ROI	*Volume mm³*	*Dice*	*Dice (Bayesian)*
Whole-cerebral-cortex (all labels combined)	253324	0.89	0.89
Cerebral-White-Matter	185615	0.92	0.91
Cerebellum-Cortex	41231	0.93	0.93
ctx-superiorfrontal	24060	0.75	0.76
Cerebellum-White-Matter	17612	0.85	0.84
ctx-inferiorparietal	15724	0.79	0.77
ctx-precentral	15576	0.76	0.74
ctx-rostralmiddlefrontal	15222	0.75	0.72
ctx-superiortemporal	13822	0.81	0.79
ctx-superiorparietal	13739	0.70	0.64
ctx-middletemporal	12339	0.74	0.72
ctx-inferiortemporal	11691	0.69	0.65
ctx-lateraloccipital	11376	0.57	0.61
ctx-postcentral	11098	0.74	0.72
ctx-supramarginal	10717	0.76	0.78
ctx-precuneus	10375	0.65	0.72
ctx-fusiform	9764	0.65	0.64
ctx-lateralorbitofrontal	8765	0.76	0.70
Brain-Stem	7528	0.54	0.65
ctx-insula	7307	0.83	0.81
ctx-caudalmiddlefrontal	6708	0.66	0.65
ctx-medialorbitofrontal	6172	0.67	0.73
ctx-lingual	5552	0.62	0.67
ctx-parsopercularis	5507	0.77	0.71
Putamen	5132	0.91	0.92
ctx-paracentral	4484	0.71	0.61
ctx-parstriangularis	4174	0.77	0.69
Caudate	3915	0.87	0.91
ctx-cuneus	3161	0.42	0.57
Pons-corticopontine-pontocerebellar-fibers	2846	0.64	0.73
ctx-caudalanteriorcingulate	2788	0.64	0.63
ctx-bankssts	2770	0.65	0.68
ctx-isthmuscingulate	2704	0.50	0.71
ctx-parsorbitalis	2625	0.45	0.43

ROI	*Volume mm^3*	*Dice*	*Dice (Bayesian)*
Pons-pontine-nuclei	2549	0.45	0.65
ctx-posteriorcingulate	2446	0.53	0.69
ctx-rostralanteriorcingulate	2362	0.67	0.68
ctx-pericalcarine	2258	0.44	0.53
ctx-entorhinal	2074	0.64	0.72
ctx-temporalpole	2071	0.60	0.55
ctx-parahippocampal	1930	0.65	0.73
PuL	1559	0.77	0.77
external-pallidum	1413	0.78	0.78
MDl	1240	0.76	0.87
ctx-frontalpole	1028	0.15	0.12
ctx-transversetemporal	935	0.61	0.63
VA	907	0.71	0.70
CA1	890	0.75	0.79
Fornix	870	0.29	0.31
Claustrum	847	0.53	0.48
Reticular	772	0.38	0.24
VPL	753	0.64	0.57
LP	697	0.65	0.74
molecular-layer-HP	673	0.48	0.50
internal-pallidum	623	0.74	0.79
subiculum	566	0.68	0.70
Dentate-cerebellum	563	0.80	0.72
alveus	502	0.50	0.58
CA4-GC-DG	483	0.70	0.76
Accumbens-area	461	0.73	0.77
Thalamus	442	0.24	0.32
Lateral-nucleus	439	0.78	0.86
CeM	393	0.50	0.67
Substancia-Nigra	376	0.56	0.70
CA2-CA3	271	0.51	0.54
AV	254	0.56	0.59
Basal-nucleus	253	0.50	0.67
SCP	243	0.59	0.72
hypothalamus-posterior	234	0.41	0.58
hypothalamus-tubular-superior	232	0.53	0.52
Accessory-Basal-nucleus	219	0.60	0.75
hypothalamus-tubular-inferior	199	0.66	0.65
PAG	177	0.59	0.77
Red-Nucleus	159	0.91	0.83
VTA	157	0.57	0.67

ROI	*Volume mm³*	*Dice*	*Dice (Bayesian)*
STN	145	0.61	0.71
Optic-Nerve	142	0.67	0.58
LGN	124	0.41	0.64
acomm	122	0.47	0.44
fimbria	111	0.42	0.26
MGN	111	0.35	0.38
VLa	109	0.34	0.33
LD	108	0.23	0.43
stria-terminals	106	0.43	0.50
Central-nucleus-of-inferior-colliculus	106	0.44	0.78
Corticoamygdaloid-transitio	101	0.50	0.58
mammillary-body	88	0.64	0.57
DR	65	0.25	0.54
Inferior-olive	65	0.05	0.26
hypothalamus-anterior-superior	57	0.59	0.55
Medial-nucleus	49	0.47	0.33
Central-nucleus	47	0.35	0.53
Anterior-amygdaloid-area-AAA	47	0.15	0.21
zona-incerta	41	0.34	0.41
hypothalamus-anterior-inferior	32	0.37	0.37
Paralaminar-nucleus	27	0.11	0.14
Cortical-nucleus	20	0.21	0.30
Rest of Hippocampus	<1	N/A	N/A
Rest of Amygdala	<1	N/A	N/A

Intra-class correlation analysis on the MIRIAD data set

Table S3: The volumes and intra-class correlation coefficients (ICC) over all the ROIs in the (simplified) NextBrain atlas for the MIRIAD data set. The ROIs are listed in from largest to smallest. The atlas simplification led to the removal of the following labels compared with the original version: 386, 401, 403, 415, 431, 436, 447, 464, 468, 469, 470, 471, 472, 499, 505, 507, 515, 520, 525, 526, 527, 528, 535, 542, 543, 546, 581, 586, 589, 591, 592, 598, 599, 600, 610, 617, 624, 630, 645, 647, 649, 655, 659, 660, 669, 671, 677, 682, 683, 685, 689, 690, 693, 694, 695, 703, 717, 724, 725, 726, 731, 755, 756, 771, 779, 827, 828, 829, 842, 844.

ROI	*Volume mm³*	*ICC*
white-matter-of-forebrain	188896.2	1.00
molecular-layer-of-pva	25508.3	1.00
granular-cell-layer-of-pva	24901.6	1.00
ctx-superiorfrontal	19807.8	0.99
ctx-rostralmiddlefrontal	17267.0	0.99
ctx-precentral	13969.5	0.98
white-matter-of-the-cerebellum	13036.5	0.98
ctx-superiorparietal	12774.2	0.97
ctx-inferiorparietal	12073.0	0.99
ctx-postcentral	11731.9	0.98
ctx-lateraloccipital	11555.3	0.99
ctx-middletemporal	11142.9	0.99
ctx-superiortemporal	11142.1	0.99
ctx-inferiortemporal	10273.6	0.99
ctx-fusiform	10019.9	0.99
ctx-supramarginal	9019.5	0.99
ctx-lateralorbitofrontal	8209.3	0.98
ctx-precuneus	6896.1	0.99
ctx-lingual	6889.9	0.98
ctx-insula	6218.0	0.99
ctx-medialorbitofrontal	5601.3	0.97
ctx-caudalmiddlefrontal	5469.2	0.98
putamen	4293.3	0.99
ctx-parsopercularis	3901.9	0.97
pons	3576.3	0.98
ctx-paracentral	3108.0	0.95
ctx-parsorbitalis	2946.3	0.96
ctx-posteriorcingulate	2738.4	0.96

ROI	*Volume mm³*	*ICC*
ctx-cuneus	2677.4	0.98
ctx-parstriangularis	2628.6	0.96
white-matter-of-hindbrain	2509.1	0.98
ctx-isthmuscingulate	2469.0	0.98
ctx-parahippocampal	2432.8	0.99
head-of-caudate	2429.2	0.99
ctx-rostralanteriorcingulate	2401.9	0.92
ctx-temporalpole	2363.0	0.97
ctx-caudalanteriorcingulate	2296.1	0.91
ctx-pericalcarine	2131.0	0.99
midbrain-(mesencephalon)	2096.2	0.96
ctx-bankssts	2007.3	0.98
ctx-entorhinal	1988.5	0.97
ctx-frontalpole	1707.0	0.94
pontine-nucleus	1173.0	0.97
ctx-transversetemporal	938.4	0.94
external-segment-of-globus-pallidus	937.7	0.98
body-of-caudate	929.3	0.96
myelencephalon-(medulla-oblongata)	927.4	0.89
medial-nucleus-of-pulvinar	612.8	0.99
rostral-subiculum	547.1	0.99
parvocellular-division-of-va	541.3	0.96
dentate-(lateral)-nucleus	514.0	0.93
lateral-posterior-nucleus-of-thalamus	494.2	0.94
claustrum	458.6	0.95
parvocellular-(central)-division-of-md	457.4	0.98
internal-segment-of-globus-pallidus	448.1	0.97
lateral-nucleus	442.7	0.96
thalamus	436.1	0.95
core-of-nucleus-accumbens	391.2	0.97
caudal-division-of-ventral-posterior-lateral-nucleus	353.6	0.95
magnocellular-(medial)-division-of-md	331.9	0.98
stratum-pyramidale-of-rostral-ca1	331.6	0.99
basolateral-nucleus-(basal-nucleus)	328.6	0.99
alveus	296.7	0.97
fornix	258.0	0.96
periaqueductal-gray-substance	250.3	0.93
multiform-(lateral)-division-of-md	248.2	0.96
magnocellular-division-of-va	242.8	0.93
stratum-lacunosum-moleculare-of-rostral-ca1	234.5	0.98
substantia-innominata	230.7	0.95

ROI	*Volume mm³*	*ICC*
reticular-nucleus-of-thalamus	225.9	0.94
anterior-nucleus-of-pulvinar	202.2	0.91
superior-cerebellar-peduncle-(brachium-conjunctivum)	186.4	0.95
ventral-division-of-basomedial-nucleus	180.6	0.99
optic-tract	170.3	0.95
lateral-nucleus-of-pulvinar	170.1	0.98
red-nucleus	169.5	0.88
molecular-layer-of-rostral-dentate-gyrus	164.9	0.99
caudal-subiculum	162.3	0.97
anteroventral-nucleus-of-thalamus	162.1	0.95
dorsal-subdivision-of-vlc	161.1	0.94
rostral-division-of-ventral-posterior-lateral-nucleus	149.2	0.89
substantia-nigra-reticular-part	137.2	0.91
stria-terminalis	132.1	0.91
ventral-tegmental-area	124.1	0.87
rostral-division-of-vl	123.6	0.91
medial-division-of-centromedian-nucleus-of-thalamus	118.7	0.91
bed-nucleus-of-stria-terminalis	118.4	0.94
substantia-nigra–compact-part	117.8	0.91
lateral-dorsal-nucleus-of-thalamus	115.6	0.92
anterior-commissure	112.4	0.97
medial-septal-nucleus	110.5	0.91
amygdalocortical-(corticoamygdaloid)-transition-area	110.3	0.99
temporal-claustrum	109.0	0.90
polyform-layer-of-rostral-dentate-gyrus	108.5	0.98
ventral-subdivision-of-vlc	107.2	0.86
stratum-pyramidale-of-caudal-ca1	101.0	0.97
stratum-pyramidale-of-uncal-ca1	99.1	0.98
anterior-amygdaloid-area	96.8	0.96
central-nucleus-of-inferior-colliculus	93.7	0.91
stratum-radiatum-of-rostral-ca1	92.6	0.98
posterior-nucleus-of-thalamus	87.0	0.92
zona-incerta	78.0	0.92
stratum-lacunosum-moleculare-of-uncal-ca1	77.6	0.98
paraventricular-nucleus	74.8	0.94
densocelllular-(paralamellar)-division-of-md	72.1	0.93
paracentral-nucleus-of-thalamus	71.7	0.93
amygdaloid-complex	70.9	0.98
tail-of-caudate	70.7	0.92
medial-subdivision-of-vlc-(area-x)	70.5	0.83
lateral-division-of-central-lateral-nucleus	67.5	0.89

ROI	*Volume mm³*	*ICC*
pyramidal-cells-of-rostral-ca4	65.2	0.99
stratum-oriens-of-rostral-ca1	62.8	0.98
inferior-cerebellar-peduncle	62.8	0.96
supraoptic-region-of-hth	61.7	0.90
lateral-olfactory-area	61.7	0.95
ventral-posterior-inferior-nucleus	59.6	0.93
laterodorsal-portion-of-sth	59.6	0.88
dorsal-lateral-geniculate-nucleus	59.2	0.93
posterior-hypothalamic-nucleus	57.0	0.86
lateral-hypothalamic-area–tuberal-part	55.1	0.94
dorsal-raphe-nucleus	53.6	0.82
lateroventral-portion-of-sth	52.5	0.85
pretectal-region	52.4	0.92
molecular-layer-of-caudal-dentate-gyrus	51.5	0.97
parafascicular-nucleus-of-thalamus	49.6	0.88
stratum-lacunosum-moleculare-of-caudal-ca1	49.4	0.97
lateral-interpositus-(emboliform)-nucleus	47.4	0.88
lateral-division-of-centromedian-nucleus-of-thalamus	46.6	0.89
mammillothalamic-tract	44.5	0.93
optic-chiasm	41.4	0.87
lateral–hypothalamic-area–posterior-part	41.1	0.89
pallidohypothalamic-area	40.8	0.93
fimbria	40.8	0.97
periventricular-area-of-thalamus	40.4	0.86
inferior-olive–principal-nucleus	38.4	0.88
uncal-subiculum	37.1	0.99
posteroventral-putamen	35.3	0.87
granular-layer-of-rostral-dentate-gyrus	33.1	0.99
inferior-nucleus-of-pulvinar	32.9	0.98
stratum-pyramidale-of-uncal-ca2	32.6	0.98
pulvinar-of-thalamus	31.8	0.97
stratum-pyramidale-of-rostral-ca3	31.8	0.98
ventral-posterior-medial-nucleus	31.2	0.85
reuniens-nucleus-(medioventral-nucleus)-of-thalamus	30.8	0.87
anterior-olfactory-nucleus	30.7	0.92
stratum-pyramidale-of-uncal-ca3	30.2	0.96
tuberomammillary-nucleus	29.2	0.92
central-part-of-medial-division-of-md	28.7	0.79
stratum-radiatum-of-caudal-ca1	28.6	0.97
ventral-pallidus	28.4	0.87
preoptic-region-of-hth	28.3	0.90

ROI	*Volume mm³*	*ICC*
stratum-lacunosum-moleculare-of-uncal-ca2	28.0	0.99
stratum-oriens-of-caudal-ca1	27.8	0.96
limitans-nucleus	27.3	0.93
centromedian-nucleus-of-thalamus	27.2	0.93
lateral-preoptic-area	26.5	0.94
polyform-layer-of-caudal-dentate-gyrus	26.0	0.97
medial-interpositus-(globose)-nucleus	25.1	0.91
stratum-radiatum-of-uncal-ca1	24.5	0.98
ventral-medial-nucleus-of-thalamus	23.3	0.92
rostral-subdivision-of-medial-nucleus	22.4	0.98
molecular-layer-of-uncal-dentate-gyrus	22.4	0.98
amygdalostriatal-transition-area	22.3	0.90
stratum-lacunosum-moleculare-of-rostral-ca3	21.5	0.97
stratum-oriens-of-rostral-ca3	19.8	0.96
medial-subdivision-of-central-nucleus	19.5	0.98
infundibular-stalk	19.5	0.88
medial-portion-of-sth	19.4	0.87
stratum-oriens-of-uncal-ca3	19.4	0.96
stratum-lacunosum-moleculare-of-rostral-ca2	19.2	0.97
amygdalohippocampal-area	18.8	0.99
pyramidal-cells-of-caudal-ca4	18.6	0.97
supraoptic-nucleus	18.3	0.92
arcuate-nucleus-of-hypothalamus	18.1	0.82
mammillary-peduncle	17.4	0.77
anteromedial-nucleus-of-thalamus	17.3	0.84
diffuse-pulvinar-nucleus	17.1	0.97
paraventricular-nucleus-of-hypothalamus	17.0	0.89
magnocellular-division-of-reticular-nucleus	16.8	0.86
diagonal-band	15.3	0.83
parvocellular-division-of-reticular-nucleus-(perireticular-nucleus)	14.9	0.81
inferior-thalamic-peduncle	14.7	0.86
ventral-subdivision-of-coa	14.4	0.98
juxtaparaventricular-lateral-hypothalamic-area	14.4	0.89
basal-nucleus-of-meynert	14.3	0.91
anterodorsal-nucleus-of-thalamus	14.2	0.83
stratum-oriens-of-uncal-ca1	14.0	0.97
stria-medullaris-of-thalamus	13.6	0.89
stratum-lacunosum-moleculare-of-uncal-ca3	13.6	0.97
septum-pellucidum	13.2	0.91
polyform-layer-of-uncal-dentate-gyrus	12.8	0.98
subparafascicular-nucleus-of-thalamus	12.6	0.86

ROI	*Volume mm³*	*ICC*
granular-layer-of-caudal-dentate-gyrus	12.6	0.97
fasciculosus-nucleus-of-thalamus	11.9	0.87
parvocellular-division-of-vpm	11.7	0.90
central-part-of-ventromedial-hypothalamic-nucleus	11.3	0.90
lenticular-fasciculus	11.0	0.61
stratum-oriens-of-uncal-ca2	11.0	0.95
medial-part-of-medial-mammillary-nucleus	10.9	0.87
layer-iii-of-piriform-cortex	10.9	0.92
stratum-pyramidale-of-caudal-ca3	10.7	0.97
stratum-radiatum-of-uncal-ca2	10.5	0.98
stratum-lucidum–of-rostral-ca3	10.4	0.97
dorsal-medial-geniculate-nucleus	10.4	0.89
paralaminar-nucleus	9.8	0.97
lateral-habenular-nucleus	9.7	0.88
stratum-pyramidale-of-rostral-ca2	9.7	0.95
parataenial-nucleus-of-thalamus	9.6	0.84
thalamic-fasciculus	9.5	0.87
dorsal-part-of-ventromedial-hypothalamic-nucleus	9.5	0.92
superficial-pulvinar-nucleus	9.2	0.93
intermediodorsal–nucleus-of-thalamus	9.1	0.80
medial-preoptic-nucleus	9.0	0.92
periventricular-nucleus–tuberal-portion	9.0	0.77
stratum-lucidum–of-uncal-ca3	8.9	0.96
basal-division-of-medial-mammillary-nucleus	8.8	0.81
stratum-lacunosum-moleculare-of-caudal-ca3	8.7	0.96
ventral-part-of-ventromedial-hypothalamic-nucleus	8.7	0.89
amygdalohippocampal-transition-area	8.5	0.98
lateral-olfactory-stria	8.2	0.80
facial-nucleus	8.1	0.86
stratum-lacunosum-moleculare-of-caudal-ca2	7.9	0.98
fastigial-(medial)-nucleus	7.9	0.80
lateral-part-of-medial-mammillary-nucleus	7.5	0.86
periventricular-nucleus–supraoptic-portion	7.3	0.68
central-gray-of-medulla-oblongata	7.1	0.83
stratum-oriens-of-caudal-ca3	7.0	0.94
lateral-tuberal-nuclei	6.8	0.91
anterior-hypothalamic-nucleus	6.6	0.91
stratum-pyramidale-of-caudal-ca2	6.5	0.97
fasciculus-retroflexus-(habenuno-interpeduncular-tract)	6.3	0.79
basal-ventral-medial-nucleus	6.3	0.91
peripeduncular-nucleus	6.0	0.80

ROI	*Volume mm³*	*ICC*
lateral-subdivision-of-central-nucleus	5.8	0.98
dorsomedial-hypothalamic-nucleus	5.7	0.91
median-preoptic-nucleus	5.2	0.77
pyramidal-cells-of-uncal-ca4	5.2	0.97
central-medial-nucleus-of-thalamus	4.8	0.70
stratum-radiatum-of-rostral-ca2	4.7	0.89
rhomboid-(central)-nucleus-of-thalamus	4.6	0.78
stratum-lucidum–of-caudal-ca3	4.0	0.97
nucleus-of-the-field-of-forel	3.9	0.91
granular-layer-of-uncal-dentate-gyrus	3.8	0.97
stratum-radiatum-of-caudal-ca2	3.7	0.97
olfactory-tubercle	3.0	0.87
supraoptic-dicussation	2.8	0.85
stratum-oriens-of-rostral-ca2	2.7	0.94
medial-habenular-nucleus	2.6	0.83
central-dorsal-nucleus-of-thalamus	2.4	0.87
dorsal-division-of-central-lateral-nucleus	2.4	0.94
stratum-oriens-of-caudal-ca2	2.1	0.93
magnocellular-(medial)-nucleus	2.0	0.89
lateral-mammillary-nucleus	1.7	0.64
supramammillary-nucleus	1.2	0.79
optic-nerve	1.0	0.29
suprachiasmatic-nucleus	1.0	0.81
habenular-commissure	0.9	0.86
interstitial-nucleus-of-cajal	0.7	0.88
medial-corticohypothalamic-tract	0.6	0.65
suprageniculate-nucleus-of-thalamus	0.5	0.74
parasubthalamic-nucleus	0.4	0.62
solitary-tract	0.3	0.76
pineal-body	0.2	0.49

Comparison of segmentation quality using non-standard MR contrast on the AHEAD dataset

To further demonstrate that the proposed approximations generalize to non-standard MR contrasts, we ran the algorithm on four randomly selected subjects on the AHEAD data set (Alkemade et al., 2020). In Figure S2, we show the segmentation results using the R1 (similar to a standard T1-weighted scan) and R2* contrasts (not similar to any standard MR contrast). As seen in the figure, especially in the close-up of the basal ganglia, the fast version of the algorithm can exploit the clear contrast of the iron rich pallidum on the R2* scans. The outline of the pallidum, including internal and external segments, is accurately captured when using the default intensity modeling settings.

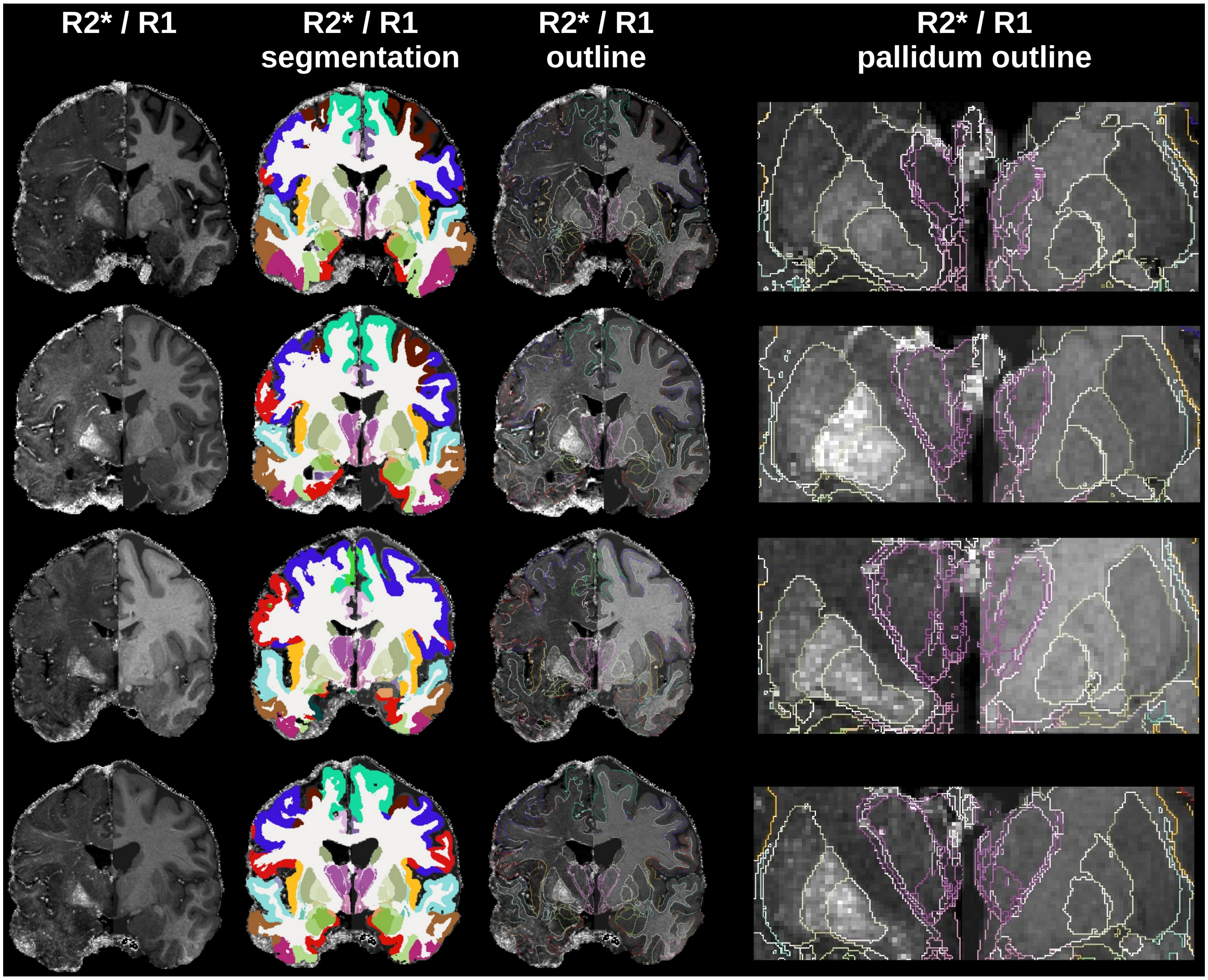

Figure S2: First column: hemispheric mosaic of R2* (left) and R1 contrast (right). Second column: automated segmentations. Third column: outline of segmentations. Rightmost column: close-up of the basal ganglia; the segmentation closely aligns to the visible contrast in the input scan, e.g., the iron-rich pallidum in R2*.
